# Comparison of TALE designer transcription factors and the CRISPR/dCas9 in regulation of gene expression by targeting enhancers

**DOI:** 10.1093/nar/gku836

**Published:** 2014-09-15

**Authors:** Xuefei Gao, Jason C.H. Tsang, Fortis Gaba, Donghai Wu, Liming Lu, Pentao Liu

**Affiliations:** 1 Wellcome Trust Sanger Institute, Hinxton, Cambridge, CB10 1HH, UK; 2 Key laboratory of Regenerative Biology, GIBH, Chinese Academy of Sciences, Guangzhou 510530, China; 3 Shanghai Institute of Immunology, Shanghai Jiaotong University School of Medicine Shanghai 200025. China; 4 Wellcome Trust-Medical Research Council Cambridge Stem Cell Institute, Universityof Cambridge, Tennis Court Rd, Cambridge CB2 1QR, UK

## Abstract

The transcription activator–like effectors (TALEs) and the RNA-guided clustered regularly interspaced short palindromic repeat (CRISPR) associated protein (Cas9) utlilize distinct molecular mechanisms in targeting site recognition. The two proteins can be modified to carry additional functional domains to regulate expression of genomic loci in mammalian cells. In this study, we have compared the two systems in activation and suppression of the Oct4 and Nanog loci by targeting their enhancers. Although both are able to efficiently activate the luciferase reporters, the CRISPR/dCas9 system is much less potent in activating the endogenous loci and in the application of reprogramming somatic cells to iPS cells. Nevertheless, repression by CRISPR/dCas9 is comparable to or even better than TALE repressors. We demonstrated that dCas9 protein binding results in significant physical interference to binding of native transcription factors at enhancer, less efficient active histone markers induction or recruitment of activating complexes in gene activation. This study thus highlighted the merits and drawbacks of transcription regulation by each system. A combined approach of TALEs and CRISPR/dCas9 should provide an optimized solution to regulate genomic loci and to study genetic elements such as enhancers in biological processes including somatic cell reprogramming and guided differentiation.

## INTRODUCTION

Transcription factors govern the stability and transition of the cellular transcriptomic network by interacting with specific genetic elements in the genome. They recruit transcription co-regulators and epigenetic modifiers to achieve orchestrated gene expression and silencing during development. To study the function of transcription factors, genetic perturbation experiments such as ectopic overexpression and depletion are commonly used. However, these approaches are insufficient in resolving the complex interaction in the native genetic context such as enhancer switching and epigenetic changes. This is exemplified in the process of reprogramming to pluripotency (1).

Ectopic expression of pluripotency factor Oct4, Sox2, Klf4 and cMyc could reprogramme differentiated MEFs to ESC-like cells (known as the induced pluripotent cells or iPSCs) with reactivation of the pluripotency network and endogenous expression of Oct4 and Nanog (1). Similarly, ectopic expression of Nanog and Klf4 have been shown to reprogramme epiblast stem cells (EpiSCs) to ESC-like cells (also known as iPSCs) (2). Compared to ESCs, which are derived from the inner cell mass of blastocyst, EpiSCs are usually derived from post-implantation embryos and dependent on Activin/FGF signalling pathway for propagation. EpiSCs are functionally pluripotent in contributing to teratoma but they are non-permissible in chemically defined media with dual ERK and GSK3 inhibition (2i) (3) and show restricted ability in chimera formation (4). Therefore, EpiSCs are regarded as existing in a more developmentally advanced ‘primed’ pluripotent state. Among the four transcription factors in reprogramming, cMyc is dispensable, and Oct4, Sox2 and Klf4 are suggested to cooperatively reactivate the pluripotency network by initiating a mesenchymal-epithelial transition and silencing of the original somatic programme through enhancer interaction in the early phase of reprogramming (5).

There has been great interest to understand the mechanism of reprogramming, but the mechanism of pluripotency locus reactivation is often difficult to discern due to non-specific or refractory reprogramming factor binding in ectopic overexpression. One avenue to study this process is through direct transcription modulation of genomic loci by designed transcription factor (dTF) engineering. Targeted dTFs can be constructed to mimic native factors in modulating expression and inducing epigenetic modification at specific regulatory element of interest (6–8). Previous successes has been reported by zinc finger protein technology (9), but wider adoption was limited. Nonetheless, the interest in dTF engineering has been recently revived due to the advance in modular assembly simplification by transcription activator-like effector (TALE) technology and the RNA-guided clustered regularly interspaced short palindromic repeat (CRISPR) system.

TALEs are natural proteins synthesized by *Xanthomonas* pathogens to activate gene expression and promote infection in plant hosts (10,11). It is later revealed that the DNA binding specificity of TALE is determined by an array of highly similar peptide repeats and specific variation of the repeat recognizes specific DNA nucleotide. This simple repeat-to-nucleotide relationship enables easy generation of artificial DNA binding domain by modular peptide repeat assembly (12,13). By fusing the tailored DNA binding domain with different effector modules, site-specific modification tools like TALE nucleases have been developed for efficient genome editing in various species (14–17). Similarly, fusion of a transcription activator or a repressor domain to designed TALE proteins has been used successfully for endogenous gene regulation in different biological processes, such as development and reprogramming (6,8,18–22). More recently, the RNA-guided CRISPR nuclease system, a natural bacterial immune response against viral pathogens (23,24), was also adapted as a genome-editing tool (25–30). In this system, the DNA binding specificity of the nuclease Cas9 protein is dependent on the simple base-pair complementarities between the engineered single guide RNA (gRNA) and its target genomic DNA sequence. Cas9 protein can be repurposed by site-specific mutations (D10A; H840A) in the nuclease domain to make the nuclease-deficient Cas9 or dCas9, which can be fused with effector domains to assemble dTF activators and repressors (21,31–34).

In this study, we set out to systematically evaluate the performance of these two rising technologies in reactivation or repression of endogenous pluripotency genes (Oct4 and Nanog) in reprogramming somatic cells or EpiSCs to iPSCs. We also attempted to identify the molecular characteristics that distinguish these two systems. We showed that the CRISPR system is as effective as or better than the TALE system in gene repression, whereas the later excels in gene reactivation in reprogramming. These differences can be, at least in part, attributed to the prominent physical binding interference of the CRISPR system. We propose that a combined application of the TALE and CRISPR system should provide an optimized approach to functionally dissect genetic elements and to regulate endogenous loci in applications such as reprogramming and stem cell differentiation.

## MATERIALS AND METHODS

### Construction of expression-ready vectors and a TALE repeat plasmid library

Plasmids containing the monomeric TALE repeat sequence of RVD variant: HD, NN, NG and NI were obtained from Zhang *et al.* (19). Monomeric repeats of each position were amplified with position-specific primers carrying corresponding linkers and BsaI cutting sites by polymerase chain reaction (PCR) (Agilent) (Supplementary Table S1 and S2). Purified PCR products were then digested by BsaI (New England BioLabs Inc?.; NEB) at 37°C for 3 h and cleaned up by PCR purification spin column (QIAGEN) before ligation by T7 ligase (Enzymatics) at 21°C for 3 h. Bands (300 bp) were then cut out and purified after gel electrophoresis. Secondary PCR with position-specific primers, TAL-F/R-assem were performed. They were then cloned into kanamycin resistant vectors by pCR-*BluntII-TOPO*^®^ PCR cloning kits (Invitrogen) and transformed by TOP10 One Shot^®^ chemically competent *Escherichia coli* (Invitrogen). Multiple colonies were randomly picked into 96-well plates for each position and sequenced. Sequencing traces were genotyped manually and correct clones were picked for expansion and archiving to create the master log. Missing triplet combinations not covered by the random assembly approach were assembled by targeted manual synthesis. Activator vectors were constructed based on the TALE architecture by Zhang *et al.* (19) and cloned into a piggyBac transposable backbone carrying a tetracycline-responsive element. The BsaI restriction site in the KRAB domain was removed and synthesized by GeneArt^®^ (Life Technologies). The VP64 transactivation domain was swapped with a KRAB repressor domain for the repressor construct. The Enhanced Green Fluorescent Protein (EGFP) reporter was also changed to a mCherry reporter. To assemble the TALE activator, bacterial clones carrying the triplet permutation at specific position and expression backbone were expanded in low salt overnight kanamycin (triplet)/ ampicillin (backbone) LB media followed by Miniprep spin column purification (QIAGEN). Eluted DNAs (50 µl) were digested for 3 h by BsaI (at 37°C, backbone)/ BsmBI (at 55°C, triplet). Digested products were then analysed by gel electrophoresis and expected bands (triplet: ∼300 bp and backbone: 7 kb) were cut out and purified with spin column (QIAGEN). Purified triplets and backbone were then ligated by T7 ligase for 1 h at 23°C. Ligated products were chemically transformed into OneShot TOP10 competent cells (Invitrogen). Clones were picked the day after and genotyped by XhoI-MluI digestion (1.7 and 2.2 kb band) and MfeI (multiple 102 bp band) digestion.

#### Plasmid vector construction

To construct PB-TRE-GC-A, PB-TRE-JW-A and PB-TRE-RJ-A, the Tetracycline Response Elements (TRE) promoter was amplified from pTight vector (Clontech) and cloned into a PB-bpA vector. cDNAs of GC-A (Addgene 47319), JW-A (Addgene 46912) and RJ-A (Addgene 48225) were cloned into the PB-TRE transposon vectors to generate different versions of PB-dCas9-A accordingly. cDNA of JW-R was ordered from Addgene (46911) and GC-R was generated by directly replacing of VP64 domain (Addgene 47319) with Krab domain at the C-terminal of the m4dCas9 protein. For PB-TRE-PL-A and PB-TRE-PL-R, two separated VP64 and KRAB domains were fused to both sides of the dCas9 coding sequence (Addgene 44246) respectively and the whole cDNAs were then cloned into PB-TRE transposon vectors. To generate the PB-U6-gRNA–EFα-mCherry-2A-rtTA-2A-BSD vector, the U6 promoter driven gRNA expression cassette (Addgene 44248) was first cloned into the PB-LTR vector. The DNA fragment encoding mCherry-2A-rtTA-2A-Blasticidin driven by the EF1α promoter was inserted at 700-bp downstream of the gRNA cassette. All sequences and maps of these constructs are available upon request. The PB-TRE-Lrh1 and PB-TRE-CKS vectors were previously described (6).

#### Preparation of MEFs for reprogramming

Mouse embryonic fibroblasts (MEFs) were prepared from 13.5-day postcoitum mouse embryos. To minimize variation among embryos, MEFs from several embryos with the same genotype were mixed together for expansion in M10 media (Dulbecco's modifiedEagle's medium (DMEM) plus 10% FBS; Fetal bovine serum). MEFs were passaged once before they were counted, divided into aliquots and cryopreserved. Approximately 1 × 10^6^ frozen MEFs were thawed and plated onto one gelatinized 15-cm tissue culture plate. MEFs were collected for electroporation at 80% confluence. M10: knockout DMEM, 10% FBS (HyClone), 1× glutamine penicillin-streptomycin (Invitrogen), and 1× NEAA (Invitrogen).

#### Transfection of MEFs and reprogramming to iPSCs

MEF transfection was performed using an Amaxa Nucleofector machine (Lonza) according to the manufacturer's protocol (program A-023). One million MEFs and 5.0 μg DNA (1.0 μg PB-transposase and 4.0 μg PB transposons) were used in each electroporation reaction. MEFs were seeded in M15 plus LIF on mitomycin-inactivated STO feeders in 10-cm dishes. Doxcycline (2.0 μg/ml) was added after transfection for transgene induction and withdrawn on day 14. iPSC colonies were selected in N2B27/2i/LIF (2i/LIF) for 10 days and stained on day 24 and expanded in standard mouse ES cell culture conditions.

#### EpiSCs culture

EpiSCs were routinely cultured in N2B27/Activin/bFGF: DMEM/F-12 (Gibco, 21331-020) medium supplemented with N2, B27, human Activin A (20 ng/ml; Peprotech) and bFGF (12 ng/ml; Invitrogen). For N2B27/2i/LIF, DMEM/F-12 (Gibco, 21331-020) medium were supplemented with N2, B27, LIF (1000 U/ml), the ERK inhibitor PD0325901 (1 μM; Stemgent) and the GSK3b inhibitor CHIRON99021 (3 μM; Stemgent). In the RT-qPCR Nanog expression analysis, *Nanog* mRNA levels were quantitated by qRT-PCR 48 h after transfection of TALE-As or dCas9-As/gRNAs and Dox induction. For EpiSC reprogramming, the culture medium was changed from N2B27/FGF/Activin to N2B27/2i/LIF (or 2i/LIF) for 14 days, 2 days after TALE-A and dCas9-A expression so as to maintain and select for induced pluripotent stem cells.

#### ChIP analysis

ES cells (10 million in one 10-cm dish) were collected 2 days after transfection of TALE or dCas9 expressing plasmids. RA-differentiated ES cells (5 million in one 15-cm dish) were collected 3 days after adding doxycycline. For validating binding of dCas9 to the targeted regions in the presence of gRNAs, ES cells were transfected with gRNA plasmids and the transfected cells stably expressing gRNAs were selected out by Blasticidin for 7 days. The Blasticidin resistant cells were subsequently transfected by the Hemagglutinin-tagged dCas9 protein expression plasmid, and were collected for crosslinking 2 days after transfection. All cells were cross-linked for 12 min by 1% formaldehyde and the crosslinking was quenched by 2.5 M glycine (0.125 M final concentration). Crosslinked cells were spun at 600 × g for 5 min, nuclei were prepared by consecutive washes with P1 buffer (10 mM Tris pH 8.0, 10 mM Ethylenediaminetetraacetic acid (EDTA) [pH 8.0], 0.5 mM EDTA, 0.25% Triton X-100) followed by P2 buffer (10 mM Tris pH 8.0, 1 mM, EDTA, 0.5 mM EGTA (Ethylene glycol tetraacetic acid), 200 mM NaCl). Pellets were resuspended in 2 ml of ChIP lysis buffer (50 mM HEPES/KOH, pH = 7.5, 300 mM NaCl, 1 mM EDTA, 1% Triton X-100, 0.1% DOC, 0.1% sodium dodecyl sulphate, protease inhibitors complete mini (Roche)) and then sonicated using BioRuptor (Diagenode) and pulsed with 15 cycles of 30 s sonication and 30 s rest. DNA was sheared to the size range between 500 and 1000 bp (confirmed on agarose gel). IgG (Cell Signalling, 2729S) and antibodies for the mKLF4 (RD, AF3158), mNANOG (Abcam, ab80892), p300 (Millipore 2031383) and H3K27ac (Abcam, ab4729) were used in ChIP analysis. Primers for qRT-PCR were used as previously reported (6).

#### Luciferase assay

Luciferase reporter plasmids (5.0 μg), TK-Renilla (0.5 μg) (Promega) were transfected into cells, together with expression vectors of the TALE or dCas9 (5.0 μg). The Oct4 luciferase assay reporter constructs carried the genomic DNA 2.4 kb upstream of the Oct4 transcription start site (TSS). The region encompasses the 1.7 kb distal and proximal enhancers and the 0.2 kb promoter. For Nanog luciferase assay reporter, the ∼1.0 kb DNA fragment of the *Nanog* 5 kb enhancer (−5145 to −4154) was cloned into a mini promoter luciferase vector. Forty-eight hours after transfection, cells were lysed with passive lysis buffer (Promega). Luciferase activities were measured with a dual-luciferase reporter assay system (Promega) according to the manufacturer's protocol.

#### Retinoic acids induced differentiation and secondary reprogramming

iPS cells produced by Dox-inducible CKS plus TALE-A or dCas9-As targeting at the Site 3–4 of the Oct4 distal enhancer were differentiated in 1.0 μM retinoic acids (RA) for 14 days. The differentiated cells were then collected and re-plated in 15-cm dish (5 million/ dish) and 6-well (1500/ well) plates for ChIP-qPCR analysis and reprogramming respectively. Secondary reprogramming was induced by adding Dox (2.0μg/ ml) again into the culture media.

#### Alkaline phosphatase staining

Cells were fixed in citrate–acetone–formaldehyde and stained using the Alkaline Phosphatase kit (Sigma-Aldrich) according to the manufacturer's instructions.

#### Flow cytometry

Flow cytometry was performed using a BD Fortessa analyser with subsequent data analysis using FlowJo 7.6.5 software. Cell sorting was performed using a MoFlo XDP (BD) cell sorter. mCherry, Green fluorescent protein (GFP) and blue fluorescent protein (BFP) were excited using 561 nm, 488 nm and 405 nm laser and detected using a 610/20, 530/30 and 440/40 filter.

#### RT-qPCR

RNA was isolated using the RNeasy Mini Kit (Qiagen). The samples were subsequently quantified and treated with gDNA Wipe-Out buffer (Qiagen). First-strand cDNA was prepared by using the QuanTect Kit (Qiagen). For each RT-PCR reaction, we used 50 to 100 ng of cDNA. Standard PCR conditions were: 94°C for 30 s, 60°C for 30 s and 68°C for 30 s for 30 cycles. For endogenous *Oct4* gene expression detection, custom designed TaqMan Gene Expression probe sets were used: forward, CTCTCCCATGCATTCAAACTGA; reverse, CCCTTGCCTTGGCTCACA; probe, CACCAGCCCTCCCT. The information of probe sets was detailed in Supplementary Table S3. All reactions were performed in a 9700HT Fast Real-Time PCR System (Applied Biosciences). Gene expression was determined relative to mouse *Gadph* using the ΔΔCt relative quantification method.

#### gRNA off target analysis

The Cas-OFFinder web tool by Bae *et al.* (35) was used to locate similar targeting sequences in the mouse genome tolerating up to three mismatches. The set of potential off-target sites were then intersected with the coordinates of the gene TSSs +/−3 kb window annotated in the Ensembl database (GRCm38.75) by bedtools (version bedtools 2-2.19.1) (36) to identify potential off-target associated genes.

#### Statistical analysis

Statistical significance was determined using a Student's *t*-test with two-tailed distribution. *P*-values <0.05 were considered as significant. Data are shown as mean and SD.

## RESULTS

### Activation of the *Oct4* and *Nanog* loci by TALE and the dCas9 activators

**Figure 1.**
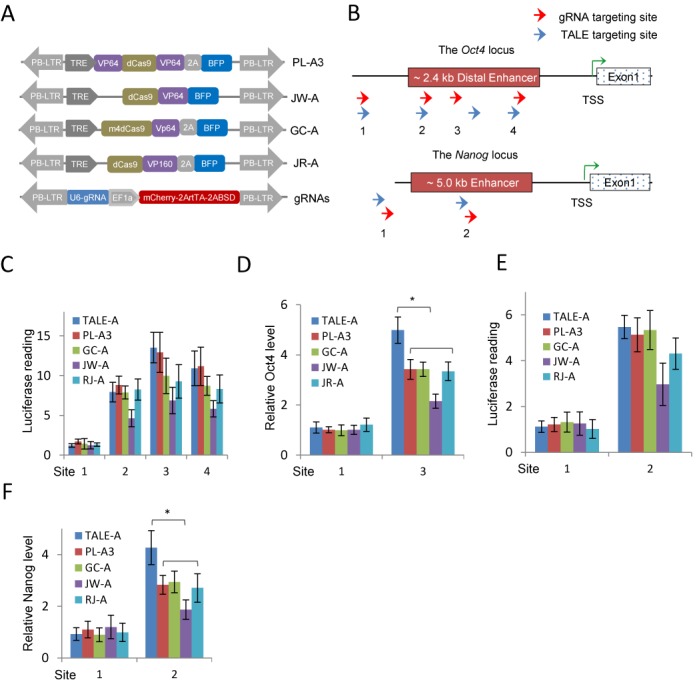
Activation of the mouse *Oct4* and *Nanog* loci by TALE-As and dCas9-As/gRNAs. (**A**) Schematic diagram of the dCas9-As evaluated in this study. In all cases, blue florescent protein (BFP) was used to track the expression of dCas9-As. gRNA expression is controlled by U6 promoter, and EF1a-mCherry is used to detect the integration of the vector into the genome. A blasticidin resistance and the reverse tetracycline transactivator (rtTA) cassettes were also linked to the mCherry cassette by T2A peptides. PB-5TR and PB-3TR are the two terminal repeat sequences of the *piggyBac* (PB) transposon. (**B**) Schematic diagram of the TALE and dCas9/gRNA targeting sites at the mouse *Oct4* and *Nanog* enhancers. Red arrows indicate the gRNA targeting sites and the blue arrows mark the TALE sites. (**C**) Activation of the *Oct4* distal enhancer luciferase reporter by the TALE-As and dCas9-As/gRNAs. (**D**) qRT-PCR analysis of the *Oct4* mRNA levels in MEFs expressing the TALE-As or dCas9-As/gRNAs. (**E**) Activation of the *Nanog* enhancer luciferase reporter by the TALE-As and dCas9-As/gRNAs. (**F**) qRT-PCR analysis of the *Nanog* mRNA levels in EpiSCs expressing the TALE-As or dCas9-As/gRNAs. All gene expression values were normalized to *Gapdh*. Results were representative of three independent experiments and were presented as ±SD, *n* = 3. *P < 0.05.

We began by comparing the ability of activating the *Oct4* locus through enhancer activation by TALE activator (TALE-A) and CRISPR/dCas9 activators (dCas9-As) in an Oct4-GFP reporter MEFs system. The transgenic Oct4-GFP MEFs contained the 18 kb fragment upstream of the Oct4 TSS and were previously shown to faithfully report the transcription status of the endogenous Oct4 locus (37). We previously showed that TALE-As targeting the distal enhancer rapidly reactivates transcription at the *Oct4* locus and is able to replace exogenous Oct4 in reprogramming MEFs to iPS cells (6). In this study, we modified the dCas9 protein (31) and made three versions of activators in which the VP64 activation domain was fused to either the N-terminal, the C-terminal or both termini of the protein. They were named as PL-A1, PL-A2 and PL-A3, respectively (Supplementary Figure S1A). We also acquired three published dCas9 activator constructs (32,33,38). They are different in the deactivating mutations of the Cas9 nuclease, in the number of activation domain repeat units and in sequential arrangement of the domains. We termed these dCas9 activators as JW-A (32), GC-A (38) and RJ-A (33), according to their origins (Figure 1A). All the dCas9 activators were cloned into a *piggyBac* transposable vector. We also linked a BFP to the dCas9 protein via the T2A peptide, and mCherry to the gRNA expression vector in order to track the expression of the system in cells (Figure 1A). The DNA targeting sequences for TALE-A (three inside and one outside the Oct4 distal enhancer) were previously described (6). To ensure comparability of TALE-As and gRNA/dCas9-As targeting sites, we constructed a hemagglutinin-tagged dCas9 vector and designed multiple gRNAs to target sequences at close proximity to the TALE-A targeting sites in the distal Oct4 enhancer and compared their binding affinity by Chip-quantitative PCR (ChIP-qPCR). We then selected four gRNA targeting sites with high binding affinity for further analysis (Figure 1B and Supplementary Figure S1B and S1C). Although the two gRNAs (g3–2 and g3–3) targeting sites overlapped with more than 12 bp with the previously validated TALE-3 targeting region, they failed to induce dCas9 binding (Supplementary Figure S1C and Supplementary Table S4).

We first investigated activation of the *Oct4* enhancer by luciferase reporter assay 48 h after transfection of TALE-A and dCas9-A/gRNA in MEFs. These luciferase constructs contain the 2.4 kb region covering all three upstream regulatory elements of the Oct4 locus (6). Out of the three dCas9-As we constructed *de novo*, PL-A3, which has a VP64 domain at both N- and C- termini of dCas9 protein, produced the highest luciferase activities (Supplementary Figure S1D), we thus used PL-A3 in all subsequent CRISPR/dCas9 experiments. The luciferase activities of dCas9-As/gRNAs were comparable to that of TALE-A targeting the same region (Figure 1C). We next examined how dCas9-As affected expression of the endogenous *Oct4* locus in MEFs by RT-qPCR. We observed the similar pattern of activation as in luciferase assay, but none of the dCas9-As activated Oct4 mRNA expression to the levels by the TALE-A (Figure 1D).

We also designed gRNA constructs and TALE-As to target the *Nanog* 5 kb upstream enhancer (Supplementary Figure 1E). Similar to the *Oct4* locus, dCas9-As could bind their targeted regions and effectively activate the luciferase reporter carrying the 5 kb upstream enhancer (Figure 1E and Supplementary Figure S1F), but again failed to achieve the same level of mRNA expression from the *Nanog* locus as the TALE-A (Figure 1F). Interestingly, we did not observe significant additive effect on *Oct4* and *Nanog* enhancer luciferase activation when we co-transfected multiple gRNAs with dCas9-A in MEFs (Data not shown).

### dCas9 activators in reprogramming somatic cells to iPS cells

**Figure 2.**
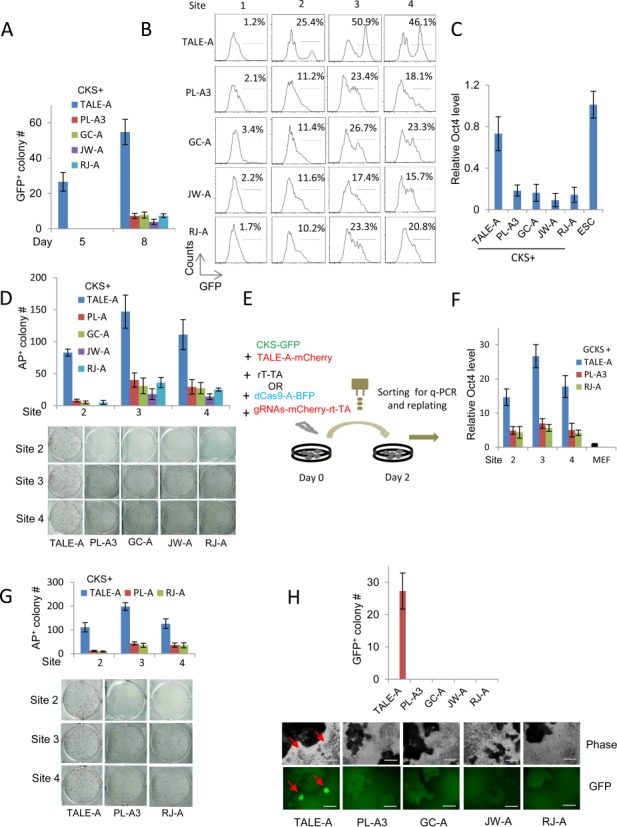
Somatic reprogramming to pluripotency by TALE-A or dCas9-As/gRNAs. (**A**) Comparison of GFP^+^ colony number reprogrammed from *Oct4-GFP* MEFs by CKS factors plus TALE-A or dCas9-As targeting at Site 3 of the *Oct4* distal enhancer on day 5 and 8 post transfection. (**B**) Flow cytometry analysis of GFP^+^ cells in *Oct4-GFP* MEFs on day 8 by CKS factors plus TALE-As or dCas9-As targeting at Sites 1–4 of the *Oct4* distal enhancer. (**C**) The endogenous *Oct4* mRNA expression levels in GFP^+^*Oct4-GFP* MEFs expressing CKS factors plus TALE-A or dCas9-As targeting at Site 3. (**D**) The number of AP^+^ colonies reprogrammed from *Oct4-GFP* MEFs as in (B). (**E**) Schematic diagram showing the design of reprogramming experiment with FACS-sorted wild-type MEFs to control for the technical variability in primary transfection. Green: GFP-tagged CKS vector, blue: BFP-tagged dCas9-As vector and red: mCherry-tagged TALE-As or gRNAs-rtTA vector. (**F**) The endogenous Oct4 expression levels in the FACS-sorted wild-type MEFs expressing the TALE-A or dCas9-As targeting at Site 2, 3 and 4 of the *Oct4* distal enhancer. (**G**) The number of AP^+^ colony reprogrammed from the FACS-sorted wild-type MEFs (20 000 cells/well) as in (F). (**H**) Reprogramming *Oct4-GFP* reporter EpiSCs by TALE-As and dCas9-As/gRNAs targeting at the Site 2 of the *Nanog* enhancer. The iPSC colonies were indicated by red arrows. The number of GFP^+^ surviving colony was quantified 14 days after 2i media selection. Scale bars: 200.0 μm. Results were representative of three independent experiments and were presented as ±SD, *n* = 3.

We previously detected GFP^Bright^ cells in *Oct4-GFP* transgene reporter MEFs after 5-day expression of ectopic reprogramming factors *Myc, Klf4* and *Sox2* (CKS) plus TALE-As targeting the Oct4 distal enhancer (6). We thus evaluated the ability of dCas9-A/gRNAs targeting the same enhancer to reprogramme the same MEFs to iPS cells. We constructed a vector that co-expresses the rtTA and gRNA cassette so that the number of vectors transfected in both the TALE-As and dCas9-A/gRNAs experiments is the same (Supplementary Figure S2A). Surprisingly, none of the dCas9-As/gRNAs produced GFP^+^ cells before day 8 (Figure 2A). Flow cytometry analysis of cells expressing TALE-As (mCherry^+^) showed that all three TALE-As targeting inside the distal enhancer (Site 2–4) produced bright GFP^+^ cells, in particular for the TALE-A targeting at the Site 3 where up to 50% of mCherry^+^ cells were GFP positive. In contrast, in cells expressing both dCas9-As/gRNAs, only GFP^dim^ cells were detected and at substantially lower percentages (Figure 2B). The discrepancy of GFP^+^ cells between dCas9-As and TALE-As was also reflected in endogenous transcription activity. RT-qPCR analysis of endogenous *Oct4* mRNA levels in the GFP^+^ cells confirmed the less effective activation of the locus by the dCas9-As at day 8 of induction (Figure 2C). After 3–4 weeks of induction, dCas9-As produced much lower numbers of alkaline phosphatase-positive (AP^+^) colonies (Figure 2D). Nevertheless, the iPSC colonies generated by the dCas9-As expressed similar levels of key pluripotency genes (Supplementary Figure S2B). To further test the reprogramming potential of the two systems in a homogenous experimental setting, we linked a GFP cassette to the CKS reading frame by 2A peptide (termed GCKS) in a *piggyBac* vector and transfected wild-type MEFs with GCKS plus either TALE-As or dCas9-As/gRNAs (Figure 2E). Two days after transfection, cells of GFP^+^/mCherry^+^ (GCKS plus TALE-As), or of GFP^+^/mCherry^+^/BFP^+^ (GCKS plus dCas9-As/gRNAs) were sorted out for RT-qPCR analysis and for subsequent reprogramming induction. We found that the Oct4 mRNA levels in TALE-As transfected MEFs were 3–4 folds higher than in cells expressing dCas9-As/gRNAs (Figure 2F), whereas the expression levels of GCKS were similar (Supplementary Figure S2C). Consistently, TALE-As targeting sites 2–4 produced 4–10 folds more colonies than their dCas9/gRNAs counterparts after 3–4 weeks continuous induction (Figure 2G).

At the *Nanog* locus, dCas9-As/gRNAs targeting the 5 kb upstream enhancer activated luciferase reporter and even increased the mRNA expression, yet they failed to produce any iPSC colonies from *Oct4-GFP* reporter EpiSCs (3) (Figure 2H). In contrast, expressing the TALE-A targeting at the Site 2, which is inside the enhancer, consistently produced iPSC colonies (Figure 2H). To confirm the inability of dCas9-As/gRNAs to reprogramme EpiSCs, we designed and tested additional four gRNAs with target sequences across the enhancer region (Supplementary Figure S1E and Supplementary Table S4). Again, these dCas9-As/gRNAs bound their target regions and induced substantial luciferase reporter activities (Supplementary Figure S1F and S2D), but no iPSC colonies were produced (data not shown).

We investigated the possibility of inadvertent off-target gene activation by dCas9-A/gRNAs, which may impede the reprogramming process. We computationally identified 58 genes that contained potential off-target binding sites 3 kb up/down stream of their TSSs (Supplementary Table S5) for all the gRNAs tested in this study. Among which, Snai1, a key mesenchymal gene, which may have blocked the mesenchymal-epithelial transition during reprogramming, was present. However, we found no induction of Snai1 mRNA expression by either Oct4 or Nanog dCas9-As/gRNAs (data not shown). Therefore, the failure of dCas9-As/gRNAs in reprogramming was unlikely to be caused by off-target gene activation.

### Less efficient epigenetic changes caused by dCas9 activators at the enhancers

**Figure 3.**
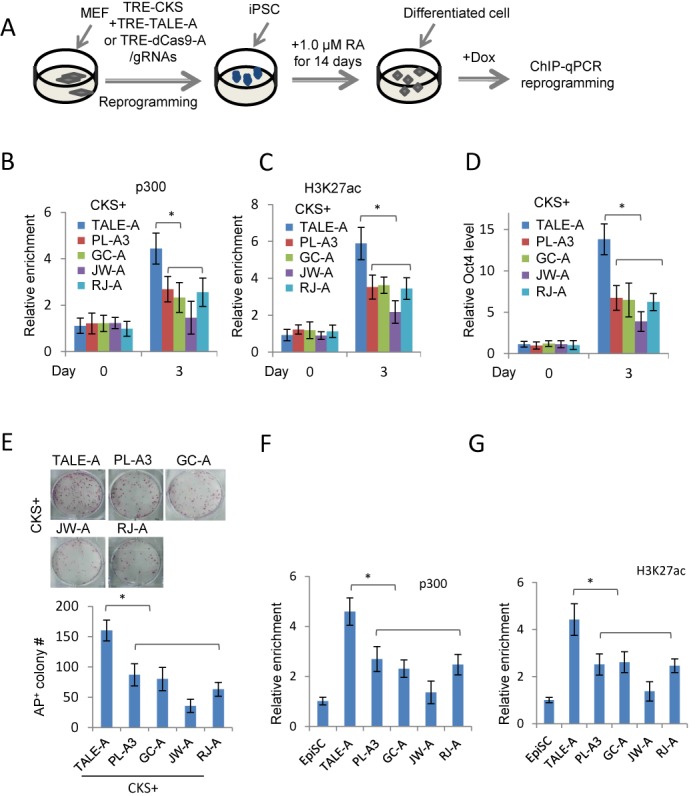
Epigenetic changes induced by TALE-As and dCas9-As/gRNAs in secondary MEF (**A**–**E**) and EpiSCs (**F** and **G**) reprogramming experiments. (A) Schematic diagram showing the experimental design of the secondary MEF reprogramming experiment. (B and C) ChIP-qPCR analysis of p300 and H3K27Ac enrichment at the *Oct4* distal enhancer. (D) The endogenous *Oct4* mRNA levels in RA-differentiated cells after 3 days of Dox induction. The relative enrichments were normalized to IgG, and a genomic region at the *Tyr* locus was used as a control region. (E) Quantification of secondary MEF reprogramming efficiency by TALE-As and dCas9-As/gRNAs. 1500 differentiated cells were plated into each well of a 6-well plate and AP^+^ colonies were scored 12 days after Dox induction. (F and G) Relative p300 and H3K27Ac enrichment at the *Nanog* enhancer in *Oct4-GFP* EpiSCs expressing TALE-A or dCas9-As targeting at the Site 2 of the *Nanog* 5 kb enhancer detected by ChIP-qPCR detection. Results were representative of three independent experiments and were presented as ±SD, *n* = 3. *P < 0.05.

We next elected to investigate the epigenetic changes at the *Oct4* distal enhancer induced by either the dCas9-A or TALE-A. It has been reported that the VP64 transactivation domain recruits activating complex component p300 and facilitates histone acetylation (39). To this end, we performed secondary reprogramming experiment using cells differentiated from iPSC clones obtained from doxycycline-inducible (Dox) CKS and dCas9-As or TALE-As (Figure 3A) to exclude the variation of transfection in primary reprogramming. These iPSCs cells contained all the reprogramming factors integrated in the genome which could be reactivated by addition of doxycycline after retinoic acid-induced differentiation. We determined the enrichment of p300 at the Site 3 after three days of Dox induction, and found that TALE-A induced significantly higher levels of p300 at the *Oct4* distal enhancer than any of the dCas9-As, detected by ChIP-qPCR (Figure 3B). Furthermore, higher levels of active histone mark H3K27Ac were induced by the TALE-A at the enhancer (Figure 3C). Similar results of p300 and H3K27Ac induction by TALE-As and dCas9-As were also found at the Site 4 (Supplementary Figure S3A and B). In agreement with the primary reprogramming experiment (Figure 2D and G), reactivation of the endogenous *Oct4* locus by dCas9-As in these differentiated cells was also significantly less efficient than the TALE-A (Figure 3D). Finally, TALE-A also outperformed all four dCas9-As (Figure 3E) in secondary reprogramming experiment.

We next examined the enrichment of H3K27Ac and p300 at the *Nanog* 5 kb enhancer region in EpiSC reprogramming, BFP^+^/mCherry^+^ and mCherry^+^ EpiSCs were FACS sorted after transfection of dCas9-As/gRNAs and TALE-As respectively. Similar to the *Oct4* distal enhancer, the TALE-A caused higher levels of p300 and H3K27Ac than dCas9-As at this enhancer. Nevertheless, even though dCas9-As failed to reprogramme EpiSCs, they were still able to induce substantial epigenetic changes at the enhancer (Figure 3F and G).

### Effective gene repression by dCas9 repressors

**Figure 4.**
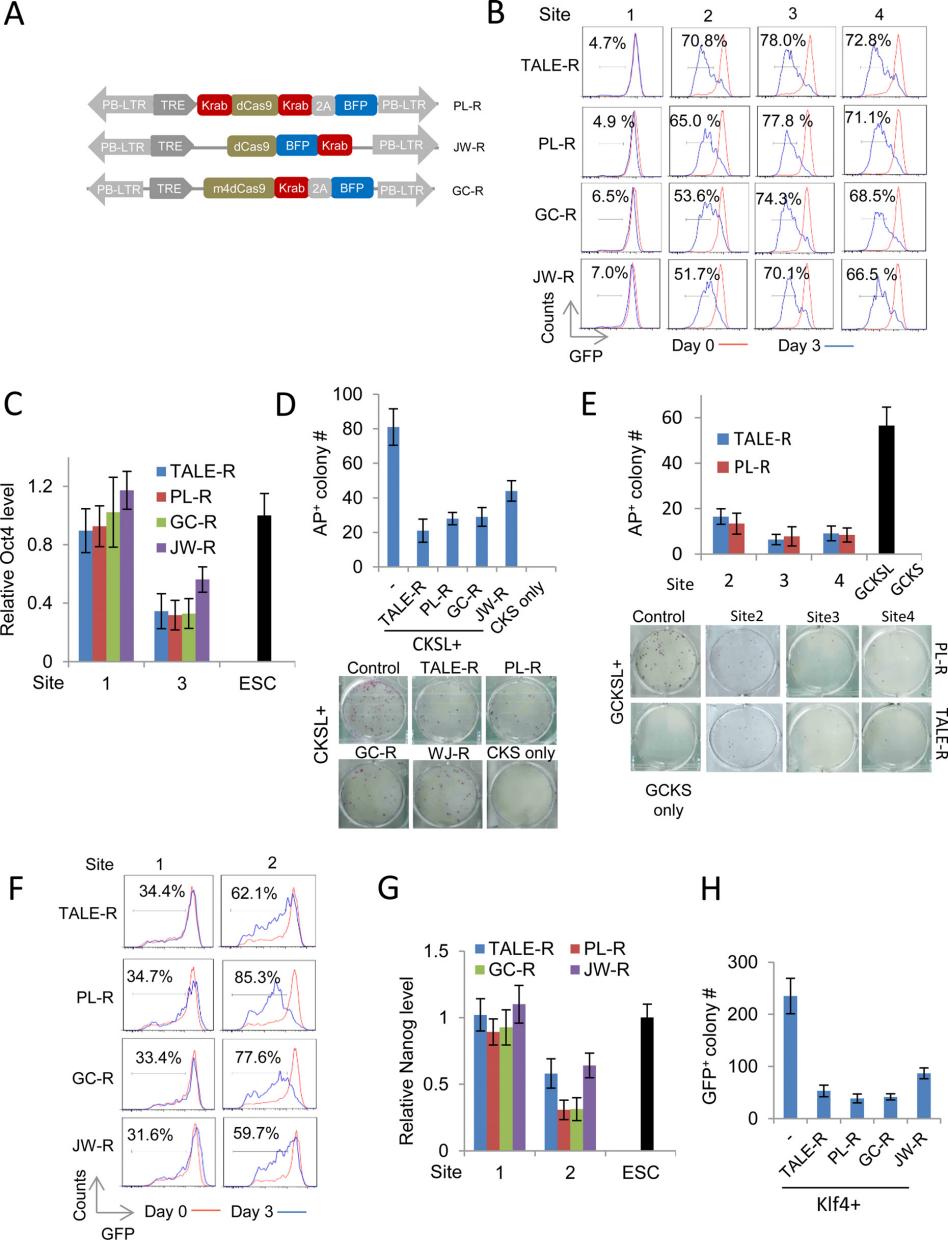
Repression of the *Oct4* and *Nanog* loci by TALE-Rs and dCas9-Rs/gRNAs. (**A**) Schematic diagram of the dCas9-R designs evaluated in this study. In all cases, blue florescent protein (BFP) was used to track the expression of dCas9-R. The gRNA vector used was the same as described in Figure 1. (**B**) Repression of the endogenous Oct4 locus in Oct4-GFP ES cells indicated by the reduction of GFP intensity in flow cytometric analysis on day 0 and day 3 of expression of the TALE-Rs or dCas9-Rs/gRNAs targeting at the Sites 1–4 of the *Oct4* distal enhancer (Gated mCherry^+^ for TALEs and mCherry^+^/BFP^+^ for dCas9-As/gRNAs). (**C**) Comparison of *Oct4* expression levels in Oct4-GFP ES cells expressing the TALE-Rs or dCas9-Rs targeting at the Site 1 or Site 3 of the *Oct4* distal enhancer by qRT-PCR. (**D**) The repressive effect of TALE-R and dCas9-Rs/gRNAs targeting at the Site 3 of the Oct4 distal enhancer on MEF reprogramming. MEFs were reprogrammed with Dox inducible CKS and Lrh1 (CKSL) factors together with the TALE-R or dCas9-R/gRNAs as in (C). ‘CKSL+’ indicates that all transfections have CKSL. ‘−’ is the CKSL only control (no repressor). ‘CKS only’ is the reprogramming negative control. (**E**) Reprogramming using FACS-sorted MEFs (as described in 2E) to control transfection variability. Wild-type MEFs were transfected and sorted on day 2 for GFP^+^/mCherry^+^ in the TALE-R transfection and for GFP^+^/mCherry^+^/BFP^+^ in dCas9-R/gRNAs (PL-R) experiments. Both TALE-Rs and dCas9-R/gRNAs (PL-R) targeted the Site 2–4 of the Oct4 distal enhancer. Sorted MEFs were re-plated (20 000 cells/ well) for reprogramming and iPSC colonies were scored by AP staining. (**F**) The repressive effect of TALE-Rs or dCas9-Rs/gRNAs targeting at the Sites 1–2 of the *Nanog* 5 kb enhancer in *Nanog-GFP* ES cells. Site 1 is located outside the enhancer region whereas Site 2 is inside. The *Nanog* repression was demonstrated by the increase of the GFP^low/dim^ fraction in Nanog-GFP ES cells. (**G**) Endogenous N*anog* mRNA levels in *Nanog-GFP* ES cells expressing the TALE-Rs or dCas9-Rs targeting at the Sites 1–2. (**H**) Repression of *Klf4-*mediated EpiSC reprogramming to iPSCs by the TALE-R and dCas9-Rs targeting at the Site 2 of the *Nanog* 5 kb enhancer. ‘Klf4+’ refers to the transfections combined with Klf4 and ‘−’ is the Klf4 control (no effector). Results were representative of three independent experiments and were presented as ±SD, *n* = 3.

To test dCas9 as repressors, we added the repressive KRAB domain to the C-terminal of the GC-dCas9 (38) and both termini of PL-dCas9 to make GC-R and PL-R. We also included a published dCas9 repressor, JW-R (32) for comparison. For all three repressors, a BFP cassette was co-expressed either through 2A peptide or direct fusion (Figure 4A). The same gRNAs that are specific to the *Oct4* distal enhancer or the *Nanog* 5 kb enhancer described above were used to guide the dCas9-Rs to their respective target sites. To compare the repression function of the two systems, the dCas9-R/gRNAs and TALE-Rs were expressed separately in either *Oct4-GFP* or *Nanog-GFP* reporter mouse ESCs (40) so that repression of Oct4 and Nanog could be conveniently tracked by GFP intensity and the number of GFP^+^ cells.

We first examined the *Oct4* locus 3 days after repressor expression. For the repressors targeting at the Site 1, which is upstream of the *Oct4* distal enhancer, neither dCas9-Rs nor TALE-R substantially affected GFP intensity or the percentage of GFP^+^ cells, indicating that the repressive function is depending on the genomic context of the targeting sequence. By contrast, all the dCas9-Rs/gRNAs and the TALE-Rs targeting at the Site 2, 3 and 4, which are within the enhancer region, efficiently suppressed GFP expression at comparable levels (Figure 4B). To quantitate the repression, cells expressing the dCas9-R/gRNAs and the TALE-Rs targeting at the Site 1 and 3 were harvested and subjected to qRT-PCR analysis. The endogenous *Oct4* mRNA was reduced to comparable levels by the dCas9-Rs/gRNAs and TALE-Rs (Figure 4C), indicating that both systems performed similarly at this enhancer.

We next tested whether the repression of the *Oct4* locus by either the TALE-R or the dCas9-R would affect MEF reprogramming. *Lrh1* was previously shown to replace exogenous Oct4 in reprogramming MEFs to iPSCs by direct binding and activating the *Oct4* locus (41). We thus co-expressed *TRE-Lrh1* with CKS (CKSL as the control) in MEFs, which eventually produced 79 AP^+^ colonies at day 25. Once the dCas9-R/gRNA or the TALE-R was co-expressed with CSKL, only fewer than 40 AP^+^ colonies were obtained (Figure 4D). Importantly, none of these colonies expressed the dCas9-R/gRNA or the TALE-R. To exclude the effects of transfection efficiency, we repeated this reprogramming experiment with GCKS and *TRE-Lrh1* combined with PL-R/gRNAs or TALE-Rs targeting at the Site 2, 3 and 4. The transfected cells were sorted out (GFP^+^/mCherry^+^ for GCKSL plus TALE-As, or GFP^+^/mCherry^+^/BFP^+^ for GCKSL plus PL-R/gRNAs) 2 days after transfection. RT-qPCR analysis showed similar induction level of LRH1 and GCKS in different transfection (Supplemental Figure S4A and S4B). PL-R targeting at all three sites inside the Oct4 distal enhancer suppressed the CKSL-induced reprogramming as efficiently as TALE-R targeting at the same sites (Figure 4E). Therefore, suppression of the *Oct4* enhancer and thus of reactivation of the locus by either dCas9-R/gRNA or the TALE-R effectively inhibited MFF reprogramming.

Besides the *Oct4* locus, we also examined the two systems in suppressing the *Nanog* locus via the 5 kb enhancer. We expressed both repression systems in *Nanog-GFP* ESCs. Effective repression of the locus was evident when GFP^+^ cells were quantitated 3 days after expression of the repressor systems. Specifically, in cells expressing GC-R and PL-R targeting at the *Nanog* enhancer Site 2, 77 and 85% cells became GFP^−^/^dim^, whereas in cells expressing the TALE-R, only 62% of them became GFP^−^/^dim^ (Figure 4F). The effective repression of the *Nanog* locus was also confirmed at the mRNA levels (Figure 4G).

We also tested the biological consequence of suppressing the *Nanog* locus in EpiSC reprogramming. Overexpression of KLF4 efficiently reprogrammes EpiSCs to iPSCs (3). We found that once the *Nanog* locus was repressed by either the dCas9-R/gRNA or the TALE-R, only very few iPSC colonies could be obtained from KLF4-mediated EpiSC reprogramming, demonstrating effective repression of the locus and the essential role of Nanog in the reacquisition of naïve pluripotency (Figure 4H).

### The dCas9/gRNA complex interferes with binding of transcription factors at enhancers

**Figure 5.**
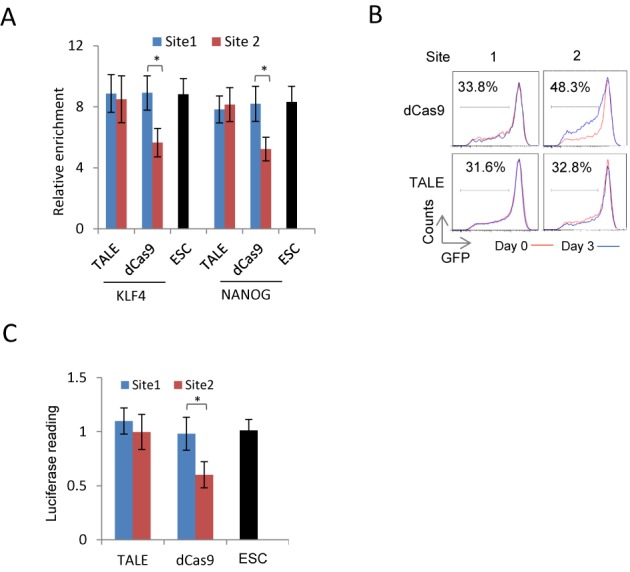
Interference of dCas9 protein on native transcription factor binding at the *Nanog* enhancer. (**A**) Change of KLF4 and NANOG binding at the 5 kb *Nanog* enhancer region induced by regulatory-domain-free dCas9 or TALE protein targeting at the Sites 1–2 detected by ChIP-qPCR in mouse ES cells. WT: ES cells transfected with an empty control vector. (**B**) Change of the GFP^low/dim^ fraction in *Nanog-GFP* ES cells induced by regulatory-domain-free dCas9 and TALE protein targeting at the Site 1–2 of the Nanog enhancer on days 0 and 3 after transfection. (**C**) Nanog enhancer luciferase reporter activities in mouse ES cells expressing regulatory-domain-free dCas9 or TALE protein targeting at the Sites 1–2. Results were representative of three independent experiments and were presented as ±SD, *n* = 3. *P < 0.05.

Comparing to the TALE proteins, the CRISPR/dCas9 system was less effective in activation but worked equally well, if not more effective, in repressing a locus. We showed in this study that one possible mechanism is the less efficient ability of dCas9-As to recruit epigenetic modifiers and co-regulator complexes. The CRISPR/dCas9 system requires a gRNA to form a complex with dCas9 at the target sites by guide RNA/targeting DNA paring, which requires local helix unwinding. This may interfere with enhancer function. In particular, it may have detrimental impact on neighbouring transcription factor binding. To address this possibility, we reviewed the ChIP-seq information of several pluripotency transcription factors, including KLF4, OCT4, NANOG and SOX2 at the *Nanog* 5kb upstream enhancer region(42) and found that the Site 2 (targeted by both TALE and dCas9) was surrounded by the predicted KLF4 and NANOG binding sites (Supplementary Figure S5). We then investigated the effect of expressing the TALE proteins or dCas9/gRNA (both without either the VP64 or KRAB domains) on the binding of NANOG and KLF4 at the *Nanog* enhancer in mouse ESCs. Expression of the TALE protein or the dCas9/gRNA that targets at the Site 1, which is outside the enhancer, did not significantly change KLF4 or NANOG binding at the enhancer detected by ChIP-qPCR (Figure 5A). On the other hand, the dCas9/gRNA targeting at the Site 2, which is within the enhancer, significantly interfered KLF4 and NANOG binding (Figure 5A). Indeed, expression of this gRNA with dCas9 (again without either VP64 or KRAB) in *Nanog-GFP* reporter ES cells substantially increased GFP^dim^ cell populations, from 33.8 to 48.3% (Figure 5B). Binding of this regulatory-domain-free dCas9/gRNA complex at the *Nanog* 5 kb enhancer also decreased luciferase reporter activities (Figure 5C). These functional consequences were not observed in ES cells expressing either the TALE protein targeting at the Site 2 or the dCas9/gRNA targeting at sequence outside the enhancer. These results showed that the dCas9/gRNA complex acted as a steric hindrance to native transcription factor binding at enhancers.

### Construction of a TALE repeat plasmid library and expression-ready vectors

In view of the distinct advantages of TALE in enhancer activation, we sought to develop a one-step process to facilitate TALE activators and repressors construction. We simplified the Golden-Gate TALE repeat assembly cloning steps into a single-step ligation reaction by constructing a plasmid library of TALE triplet repeat. Residue variable di-residues: HD, NI, NG and NN were chosen to target nucleotide cytosine, adenosine, thymine and guanine for library construction. A final 18-mer-long TALE repeats assembly design was chosen for this library and it is constructed by ligating six triplet-inserts, each carrying position-specific linkers, to an expression-ready activator/repressor backbone. Our previous report showed that the length of the TALE repeats affects the modulation efficiency and an array of 18-mer appears to be an optimal balance of targeting specificity and assembly complexity (6). In total, 384 (i.e. 6 × 64) unique triplet plasmids were cloned to cover all the possible permutations of an 18-mer-long TALE repeat by randomly ligating a pool of RVD monomers amplified with primers carrying position-specific linkers. Multiple colonies were then picked and sequenced to confirm all the possible permutations. We evaluated the assembly efficiency by constructing 10 additional TALEs, and the average success rate is 73.8% (50–100%). Luciferase reporter activation and MEF reprogramming experiment validated the TALEs constructed by this platform (Supplementary Figure S6A–D).

## DISCUSSION

Designer transcription factors are valuable tool for investigating the biological function of particular gene and cellular transcription regulatory network. Several platforms are currently available to allow engineering of transcription factor for transcription modulation and epigenetic modification at specific genetic locus (43–46). The TALE system has the advantage of customizable length of DNA binding domain. TALEs are also natural transcription factors evolved in *Xanthomonas* for host plant transcription regulation. On the other hand, it is easier to target multiple genomic loci simultaneously by the CRISPR system. Importantly, it is more scalable and cost-effective due to the simplicity and availability of oligonucleotide synthesis service. Recently, a number of groups have reported the use of gRNA libraries for genome-wide gene knockout screens, highlighting the power of this technology (47,48). Systematic evaluation of the TALE and CRISPR system for transcription regulation is however lacking.

We compared the two systems at multiple levels of both transcription activation and repression of the Oct4 and Nanog loci, including *in vitro* luciferase reporter activities, transcription levels from the endogenous loci and cellular reprogramming. This comprehensive comparison revealed several important findings that should guide the future application of these two platforms.

Firstly, our data clearly showed that there is a discrepancy between luciferase reporter assay and endogenous locus reactivation or cellular reprogramming. TALE-As were able to achieve good activation activity in all three assays, while dCas9-As failed to produce any iPSC colonies despite successful mRNA expression reactivation and similar activation in luciferase reporter assays. This result is not unexpected, as these three assays represented evaluation of increasing stringency. Activation of luciferase reporter by designer transcription factor is less demanding than endogenous mRNA reactivation, as the exogenous reporter is not subjected to local epigenetic modifications such as heterochromatin formation and promoter methylation. Nevertheless, we believe that cellular reprogramming provides a more meaningful and realistic assessment of the efficiency of gene reactivation by designer transcription factors, in particular if the technology is to suit its unique application in studying biological processes such as enhancer usage and gene reactivation mechanism in cell fate decision. The inefficiency of dCas9 in producing iPSCs despite successful reactivation of gene expression suggested that the dCas9 protein might interfere with the stabilization of the reactivated locus in later stages of reprogramming or insufficient levels of reactivation at reprogramming initiation. These speculations were supported by lower levels of mRNA reactivation compared to TALE-As and the interference of local NANOG and KLF4 binding at the Nanog enhancer in ESCs.

Secondly, we demonstrated that both the TALE and the dCas9 activators have preferential activity when targeted to enhancers but not to regions outside enhancers. Targeting sites outside the enhancer region consistently failed to substantially activate or repress transcription. This is likely due to context dependent recruitment of transcription co-regulators by the activators or repressors, where gene expression regulation at normal biological condition is also mediated by synergistic binding of multiple transcription factors at defined ‘enhanceosome’ regions (42). The genetic context of the targeting site therefore has huge impact on the success of transcription modulation of the gene of interest. We have also compared different dCas9 effector designs in our study. The performance of all the designs are generally similar, but we noticed that the architecture by Gilbert *et al*. (JW-A/R) (32) is less potent in both genetic activation and repression. It can be due to the direct fusion of the BFP with the activator and repressor domain, which may compromise the presentation and/or recruitment of co-regulatory complexes.

Our data also consistently revealed that TALE-As are superior to the dCas9-As in transcription activation both in *in vitro* assays and in reprogramming both MEFs and EpiSCs to iPSCs. dCas9-As are less efficient in recruiting transcription coregulators p300 and inducing H3K27Ac histone modifications at their targeting sites. This is unlikely caused by the configuration of the effector domain since the activation potency did not correlate with the number or position of VP16 transactivation domain repeats in the dCas9 protein. In contrast to the limitation in gene activation, the dCas9-Rs showed comparable or better gene repression than the TALE-Rs. The effective gene repression by dCas9-Rs and its low potency in activating genomic loci suggested that the system might have certain intrinsic molecular characteristics that negatively affect endogenous transcription. The dCas9 proteins alone, without any added functional domains such as VP64 or KRAB, can physically interfere binding of native transcription factors KLF4 and NANOG at the *Nanog* enhancer. This interference can be detrimental to endogenous gene expression as these native TFs are required for expression regulation and long-range interaction (49). It remains to be determined whether the dCas9/gRNA complex interference is caused by direct physical blocking of native transcription factor binding motif by the dCas9 protein bulk or by local DNA duplex configuration change by invading gRNA–DNA pairing. Regardless, these results provided the scientific justification of a combined TALE/dCas9 approach for efficient simultaneous genetic activation and repression of independent genomic loci.

In summary, we have comprehensively compared the TALE and dCas9 systems in regulating expression of genomic loci. Our findings have identified preferential applications and the underlying mechanistic rationale of individual systems in transcription modulations. The information and tools presented in this study provide a valuable resource and can facilitate future applications of the two systems in dissecting complex biological processes such as guided stem cell differentiation and cell lineage reprogramming.

## SUPPLEMENTARY DATA

Supplementary Data are available at NAR Online.

SUPPLEMENTARY DATA
