# Supplementary material for: Comparison of TALE designer transcription factors and the CRISPR/dCas9 in regulation of gene expression by targeting enhancers
Source: Nucleic Acids Res. 2014 Sep 15;42(20):e155. doi: 10.1093/nar/gku836 (PMC4227760; doi:10.1093/nar/gku836)
Supplement: SUPPLEMENTARY DATA [file supp_42_20_e155__index.html]

Comparison of TALE designer transcription factors and the CRISPR/dCas9 in regulation of gene expression by targeting enhancers — Comparison of TALE designer transcription factors and the CRISPR/dCas9 in regulation of gene expression by targeting enhancers — SUPPLEMENTARY DATA 

# Comparison of TALE designer transcription factors and the CRISPR/dCas9 in regulation of gene expression by targeting enhancers

## SUPPLEMENTARY DATA

**Files in this Data Supplement:**

- SUPPLEMENTARY DATA
- SUPPLEMENTARY DATA
- SUPPLEMENTARY DATA
- SUPPLEMENTARY DATA
