# Supplementary material for: Comparison of TALE designer transcription factors and the CRISPR/dCas9 in regulation of gene expression by targeting enhancers
Source: Nucleic Acids Res. 2014 Sep 15;42(20):e155. doi: 10.1093/nar/gku836 (PMC4227760; doi:10.1093/nar/gku836)
Supplement: SUPPLEMENTARY DATA [file supp_gku836_nar-01182-met-h-2014-File003.docx]

**Supplementary Figure legends:**

**Supplementary Figure 1**. (A) Schematic diagram of the three dCas9-A vectors constructed *de novo* in this study. The VP64 domain was fused to the N-terminal and C-terminal or both sides of dCas9 protein to construct vectors PL-A1, PL-A2 and PL-A3. (B) The targeting sequences of dCas9-As/gRNAs and TALE-As in the Oct4 distal enhancer region. dCas9-As/gRNAs targeting sequences were highlighted in red and the PAM region were underlined, the TALE targeting sequences were highlighted in blue and the overlapping sequences were highlighted in green. (C) Validation of dCas9 protein binding to the targeted regions in the presence of gRNAs by ChIP-qPCR. Hemagglutinin tags were fused to the two ends of the dCas9 protein (shown in bottom) to allow immunoprecipitation and regional binding affinity quantification by qPCR. An unrelated gRNA was used as the control. (D) Activation of the Oct4 distal enhancer luciferase reporter by PL-A1, A2 and A3. Only the gRNA targeting at the Site 3 activates the reporter. (E) The targeting sequences of dCas9-As and TALE-As in the Nanog -5kb enhancer. (F) Validation of dCas9 binding to the targeted regions in the Nanog enhancer in the presence of gRNAs by ChIP-qPCR assay. The same control was used as in (D). Results were representative of three independent experiments and were presented as mean ± SD, n =3.

**Supplementary Figure 2.** (A) Schematic diagram listing the vectors used in the MEF reprogramming experiments: PB: Piggybac transposon flanked; TRE: Tetracycline-responsive element; CAG: Cytomegalovirus early enhancer-chicken beta-actin-rabbit beta-globin fusion promoter; rtTA: reverse tetracycline transactivator; BFP: Blue fluorescence protein; EF1a: Human elongation factor-1 alpha promoter. The rtTA cassette was fused with the gRNA expression vector in the dCas9-As transfection, while it is separated in the TALE-As transfection. (B) The expression levels of several key pluripotency genes in iPSCs reprogrammed by CKS plus dCas9-As/gRNAs targeting Site 3 of the Oct4 distal enhancer. (C) The expression levels of exogenous GCKS in FACS-sorted wild type MEFs expressing GCKS factors plus the TALE-A or dCas9-As targeting Sites 2-4 of the Oct4 distal enhancer. (D) Activation of the *Nanog* 5kb enhancer luciferase assay reporter by PL-As with gRNAs targeting at sequences located either inside or outside the enhancer region. Results were representative of three independent experiments and were presented as mean ± SD, n =3.

**Supplemental Figure 3.** (A) and (B) ChIP-qPCR analysis of p300 and H3K27Ac enrichment at the *Oct4* distal enhancer in secondary reprogramming experiment at day 0 and 3 after Dox induction. The TALE-A and dCas9-As/gRNAs targeted Site 4 of the Oct4 distal enhancer. The relative enrichments were normalized to IgG, and a genomic region at the *Tyr* locus was used as the unrelated control. Results were representative of three independent experiments and were presented as mean ± SD, n =3.

**Supplemental Figure 4.** (A) and (B) The expression levels of exogenous GCKS and Lrh1 in FACS-sorted wild type MEFs after transfection of the GCKS and Lrh1 expression vectors with either TALE-R or PL-R/gRNAs targeting Sites 2-4 of the Oct4 distal enhancer. Results were representative of three independent experiments and were presented as mean ± SD, n =3.

**Supplemental Figure 5.** Genome browser representations of the ChIP-seq binding profiles of NANOG and KLF4 at the *Nanog* locus. The predicted binding sites of NANOG and KLF4 and the targeting Sites 2 (marked with red asterisk) of TALE and dCas9/gRNA were indicated.

**Supplementary Figure 6.** (A) Schematic diagram illustrating the design of the TALE plasmid library. Triplet inserts were cloned with specific position linkers in each position. The workflow on the right described the steps of generating TALE-As starting from the bacterial clones of TALE repeats. The estimated hands-on time were indicated in italics. The bottom panel showed the architecture of the construct, PB: PiggyBac, N: N-terminal, C: C-terminal, TRE: Tetracycline (On) responsive element, bpA: poly-A signal sequence. (B) Validation of the one-step TALE assembly library by Oct4 distal enhancer luciferase reporter activation in MEFs. The targeting sequences were the same as the Site 3 of the *Oct4* distal enhancer. TALE-18 was assembled by the TALE-repeat library with 18 TALE-repeats in the DNA binding domain while TALE-24 was constructed by hierarchical Golden-Gate cloning as previously described. (C) Microscopic pictures of iPSC colonies reprogrammed from *Zfp42(Rex1)-GFP* reporter MEFs (Guo et al., 2011) with Dox inducible CKS factor plus TALE-18. Rex1 is only expressed in ES cells so Rex1-GFP provides a convenient way to monitor reprogramming. Scale bars: 200.0 μm. (D) Expression of pluripotency markers in iPSC clones reprogrammed from *Zfp42-GFP* reporter MEFs with CKS factor plus TALE-18. All iPSCs and mouse ESCs were cultured in N2B27/2i/LIF. ES cell control was AB2.2. Results were presented as mean ± SD, n=3.

**Supplementary Reference:**

[Guo G](http://www.ncbi.nlm.nih.gov/pubmed?term=Guo%20G%5BAuthor%5D&cauthor=true&cauthor_uid=21533166), [Huang Y](http://www.ncbi.nlm.nih.gov/pubmed?term=Huang%20Y%5BAuthor%5D&cauthor=true&cauthor_uid=21533166), [Humphreys P](http://www.ncbi.nlm.nih.gov/pubmed?term=Humphreys%20P%5BAuthor%5D&cauthor=true&cauthor_uid=21533166), [Wang X](http://www.ncbi.nlm.nih.gov/pubmed?term=Wang%20X%5BAuthor%5D&cauthor=true&cauthor_uid=21533166), [Smith A](http://www.ncbi.nlm.nih.gov/pubmed?term=Smith%20A%5BAuthor%5D&cauthor=true&cauthor_uid=21533166). A PiggyBac-based recessive screening method to identify pluripotency regulators. [PLoS One.](http://www.ncbi.nlm.nih.gov/pubmed/?term=A+PiggyBac-based+recessive+screening+method+to+identify+pluripotency) 2011 Apr 18;6(4):e18189. doi: 10.1371/journal.pone.0018189.
