# Supplementary material for: Comparison of TALE designer transcription factors and the CRISPR/dCas9 in regulation of gene expression by targeting enhancers
Source: Nucleic Acids Res. 2014 Sep 15;42(20):e155. doi: 10.1093/nar/gku836 (PMC4227760; doi:10.1093/nar/gku836)
Supplement: SUPPLEMENTARY DATA [file supp_gku836_nar-01182-met-h-2014-File004.pptx]

## Slide 1
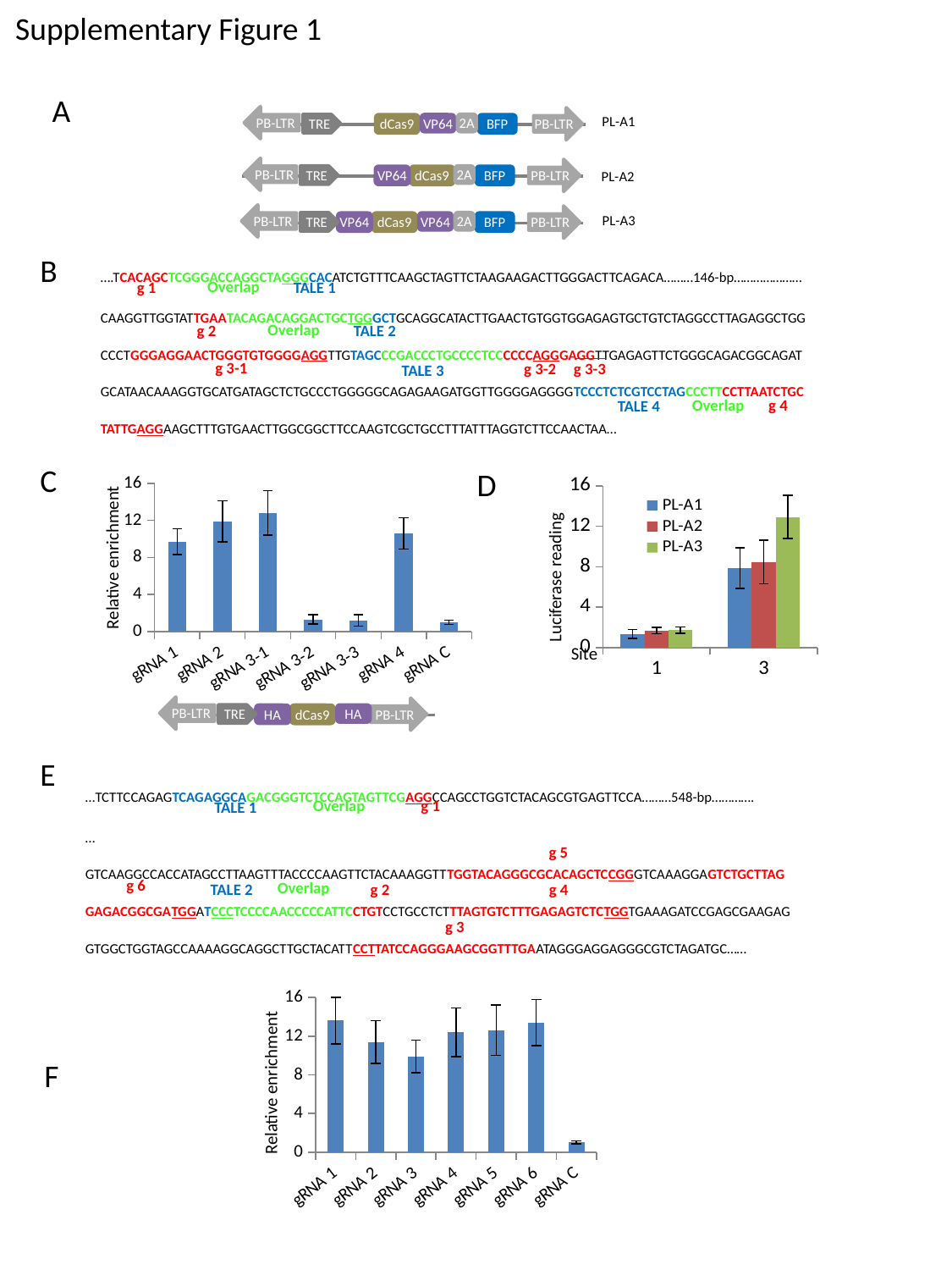

Supplementary Figure 1
A
PL-A1
PB-LTR
PB-LTR
2A
TRE
VP64
dCas9
BFP
PB-LTR
PB-LTR
PL-A2
2A
TRE
VP64
dCas9
BFP
PB-LTR
PL-A3
PB-LTR
2A
TRE
VP64
VP64
dCas9
BFP
….TCACAGCTCGGGACCAGGCTAGGGCACATCTGTTTCAAGCTAGTTCTAAGAAGACTTGGGACTTCAGACA………146-bp…………………
CAAGGTTGGTATTGAATACAGACAGGACTGCTGGGCTGCAGGCATACTTGAACTGTGGTGGAGAGTGCTGTCTAGGCCTTAGAGGCTGGCCCTGGGAGGAACTGGGTGTGGGGAGGTTGTAGCCCGACCCTGCCCCTCCCCCCAGGGAGGTTGAGAGTTCTGGGCAGACGGCAGATGCATAACAAAGGTGCATGATAGCTCTGCCCTGGGGGCAGAGAAGATGGTTGGGGAGGGGTCCCTCTCGTCCTAGCCCTTCCTTAATCTGCTATTGAGGAAGCTTTGTGAACTTGGCGGCTTCCAAGTCGCTGCCTTTATTTAGGTCTTCCAACTAA…
B
Overlap
g 1
TALE 1
Overlap
g 2
TALE 2
g 3-1
g 3-3
g 3-2
TALE 3
Overlap
g 4
TALE 4
### Chart
| Category | PL-A1 | PL-A2 | PL-A3 |
|---|---|---|---|
| 1 | 1.3431 | 1.688 | 1.711 |
| 3 | 7.8371 | 8.452 | 12.91521 |Luciferase reading
Site
C
D
### Chart
| Category | |
|---|---|
| gRNA 1 | 9.7 |
| gRNA 2 | 11.9 |
| gRNA 3-1 | 12.8 |
| gRNA 3-2 | 1.3 |
| gRNA 3-3 | 1.2 |
| gRNA 4 | 10.6 |
| gRNA C | 1.0 |Relative enrichment
PB-LTR
PB-LTR
TRE
HA
HA
dCas9
E
…TCTTCCAGAGTCAGAGGCAGACGGGTCTCCAGTAGTTCGAGGCCAGCCTGGTCTACAGCGTGAGTTCCA………548-bp………….
…GTCAAGGCCACCATAGCCTTAAGTTTACCCCAAGTTCTACAAAGGTTTGGTACAGGGCGCACAGCTCCGGGTCAAAGGAGTCTGCTTAGGAGACGGCGATGGATCCCTCCCCAACCCCCATTCCTGTCCTGCCTCTTTAGTGTCTTTGAGAGTCTCTGGTGAAAGATCCGAGCGAAGAGGTGGCTGGTAGCCAAAAGGCAGGCTTGCTACATTCCTTATCCAGGGAAGCGGTTTGAATAGGGAGGAGGGCGTCTAGATGC……
g 5
g 6
Overlap
TALE 2
g 2
g 4
g 3
Overlap
g 1
TALE 1
### Chart
| Category | |
|---|---|
| gRNA 1 | 13.6 |
| gRNA 2 | 11.4 |
| gRNA 3 | 9.9 |
| gRNA 4 | 12.4 |
| gRNA 5 | 12.6 |
| gRNA 6 | 13.4 |
| gRNA C | 1.0 |Relative enrichment
F

## Slide 2
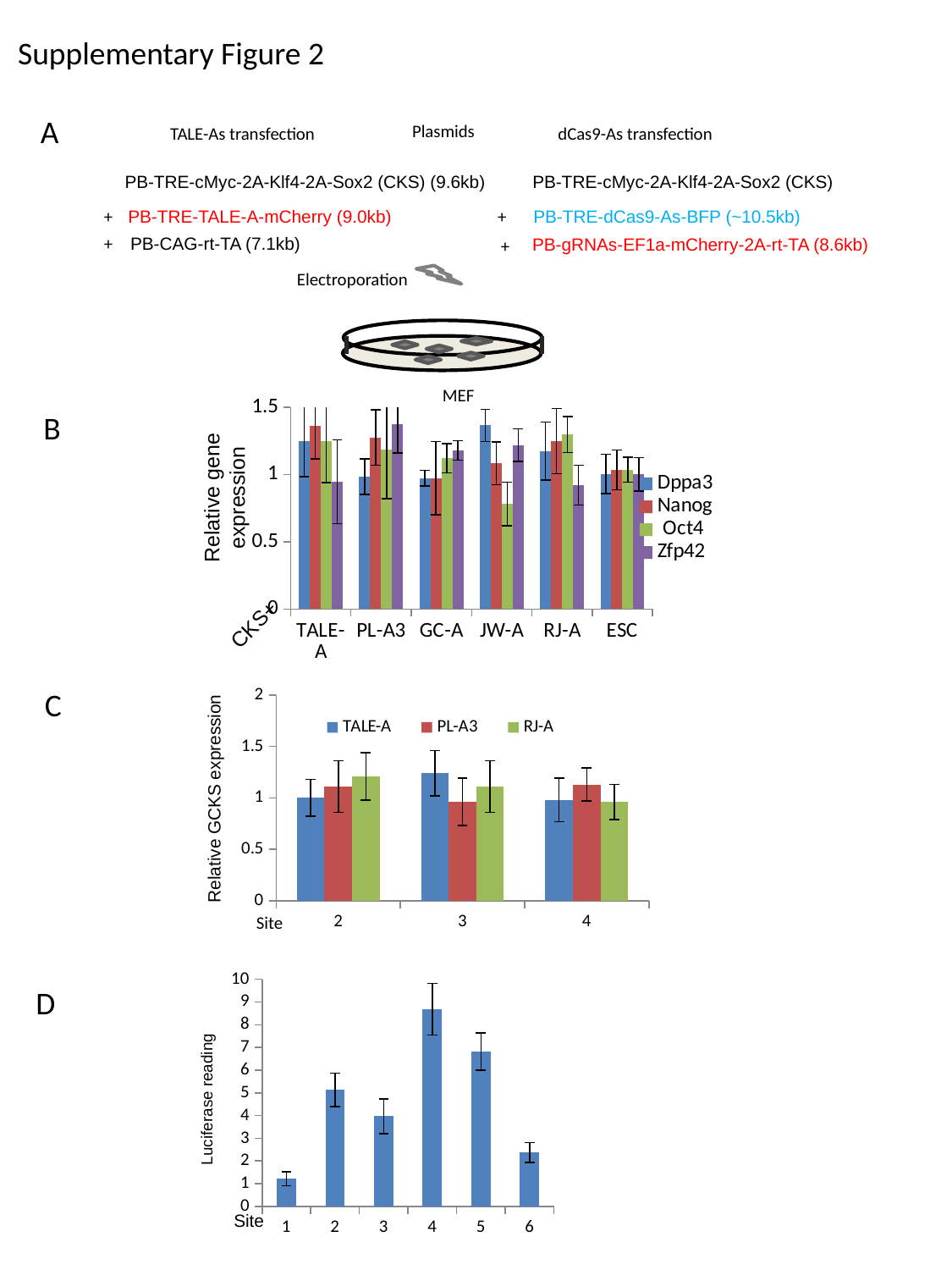

Supplementary Figure 2
A
Plasmids
TALE-As transfection
dCas9-As transfection
PB-TRE-cMyc-2A-Klf4-2A-Sox2 (CKS)
PB-TRE-cMyc-2A-Klf4-2A-Sox2 (CKS) (9.6kb)
+
PB-TRE-TALE-A-mCherry (9.0kb)
+
PB-TRE-dCas9-As-BFP (~10.5kb)
+
PB-CAG-rt-TA (7.1kb)
PB-gRNAs-EF1a-mCherry-2A-rt-TA (8.6kb)
+
Electroporation
### Chart
| Category | Dppa3 | Nanog | Oct4 | Zfp42 |
|---|---|---|---|---|
| TALE-A | 1.24732141 | 1.36413241 | 1.247291432 | 0.94739121 |
| PL-A3 | 0.98423109 | 1.27429813 | 1.18423109 | 1.374219 |
| GC-A | 0.97412891 | 0.97438291 | 1.1217912 | 1.1795321 |
| JW-A | 1.3648711 | 1.08413293 | 0.78174293 | 1.2179804312 |
| RJ-A | 1.17412309 | 1.2483129 | 1.29812681 | 0.9219234 |
| ESC | 1.0041432 | 1.034103 | 1.035431 | 1.0026814 |Relative gene expression
CKS+
MEF
B
### Chart
| Category | TALE-A | PL-A3 | RJ-A |
|---|---|---|---|
| 2 | 1.0 | 1.11 | 1.21 |
| 3 | 1.24 | 0.96 | 1.11 |
| 4 | 0.98 | 1.13 | 0.96 |Relative GCKS expression
Site
C
### Chart
| Category | |
|---|---|
| 1 | 1.212 |
| 2 | 5.13 |
| 3 | 3.97 |
| 4 | 8.68 |
| 5 | 6.82 |
| 6 | 2.37 |Luciferase reading
Site
D

## Slide 3
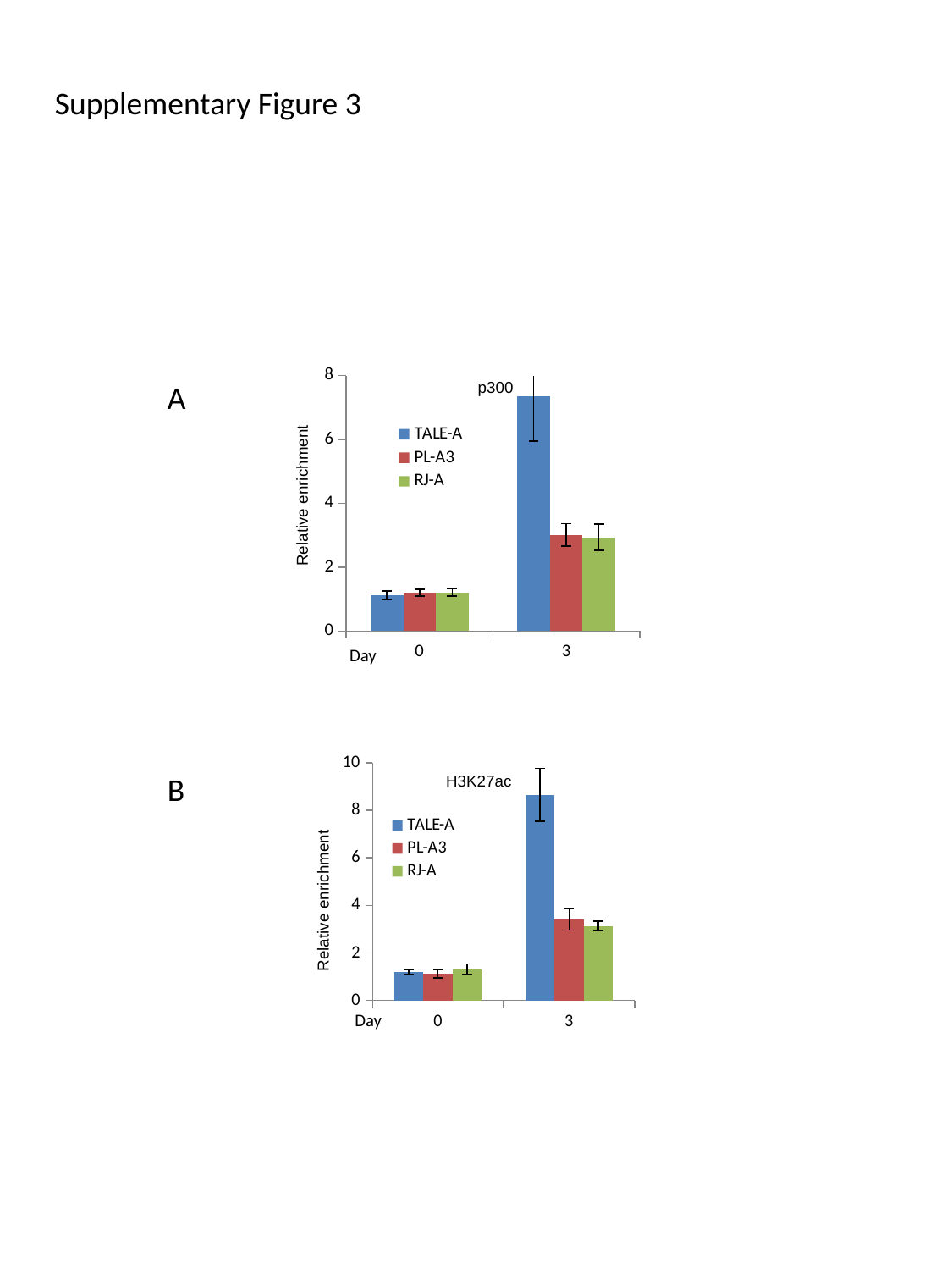

Supplementary Figure 3
### Chart
| Category | TALE-A | PL-A3 | RJ-A |
|---|---|---|---|
| 0 | 1.12 | 1.2 | 1.21 |
| 3 | 7.35 | 3.01 | 2.93 |A
p300
Relative enrichment
Day
### Chart
| Category | TALE-A | PL-A3 | RJ-A |
|---|---|---|---|
| 0 | 1.2 | 1.12 | 1.32 |
| 3 | 8.65 | 3.41 | 3.13 |Relative enrichment
Day
H3K27ac
B

## Slide 4
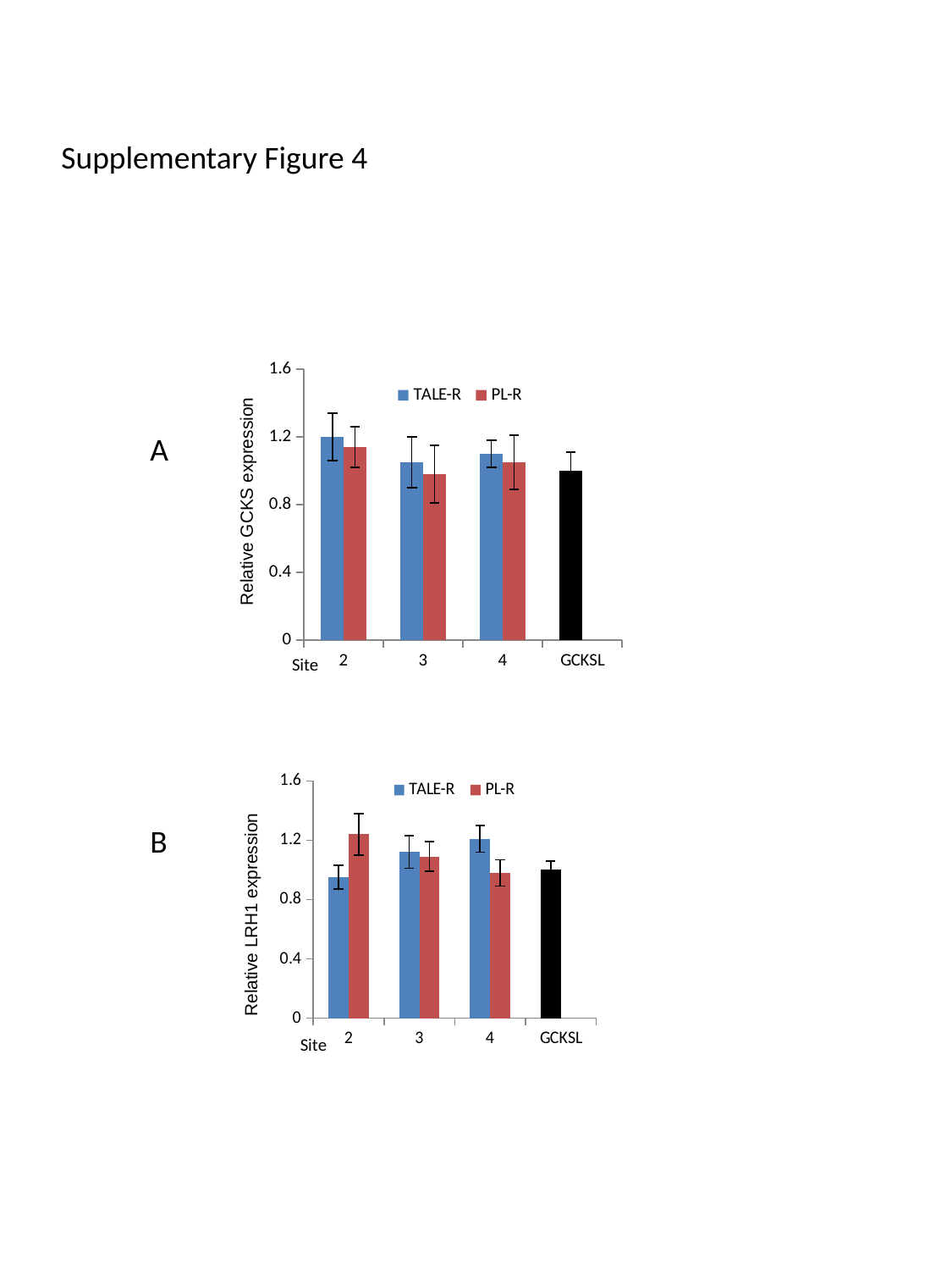

Supplementary Figure 4
### Chart
| Category | TALE-R | PL-R |
|---|---|---|
| 2 | 1.2 | 1.14 |
| 3 | 1.05 | 0.98 |
| 4 | 1.1 | 1.05 |
| GCKSL | 1.0 | None |Relative GCKS expression
A
Site
### Chart
| Category | TALE-R | PL-R |
|---|---|---|
| 2 | 0.95 | 1.24 |
| 3 | 1.12 | 1.09 |
| 4 | 1.21 | 0.98 |
| GCKSL | 1.0 | None |B
Relative LRH1 expression
Site

## Slide 5
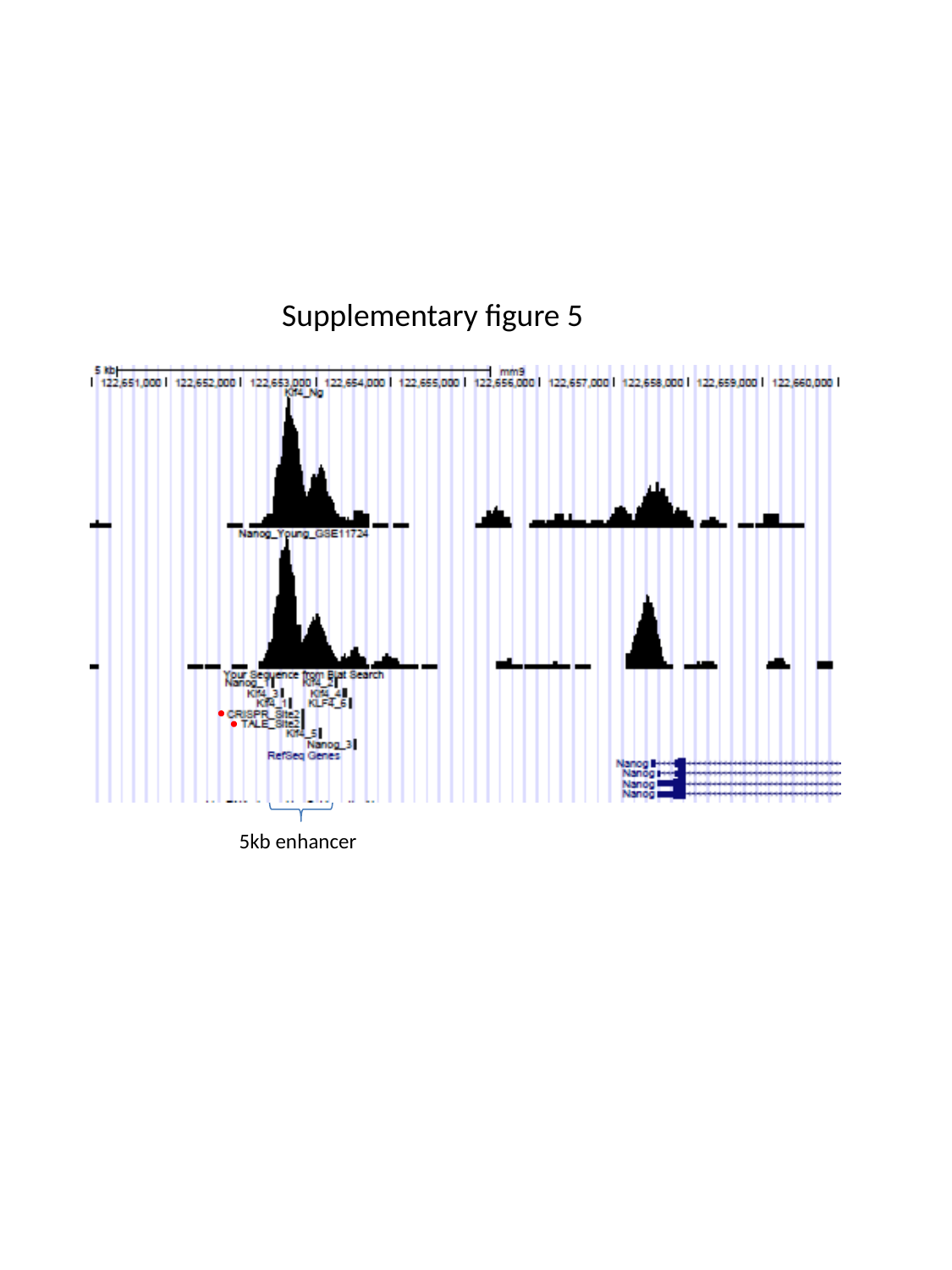

Supplementary figure 5
5kb enhancer

## Slide 6
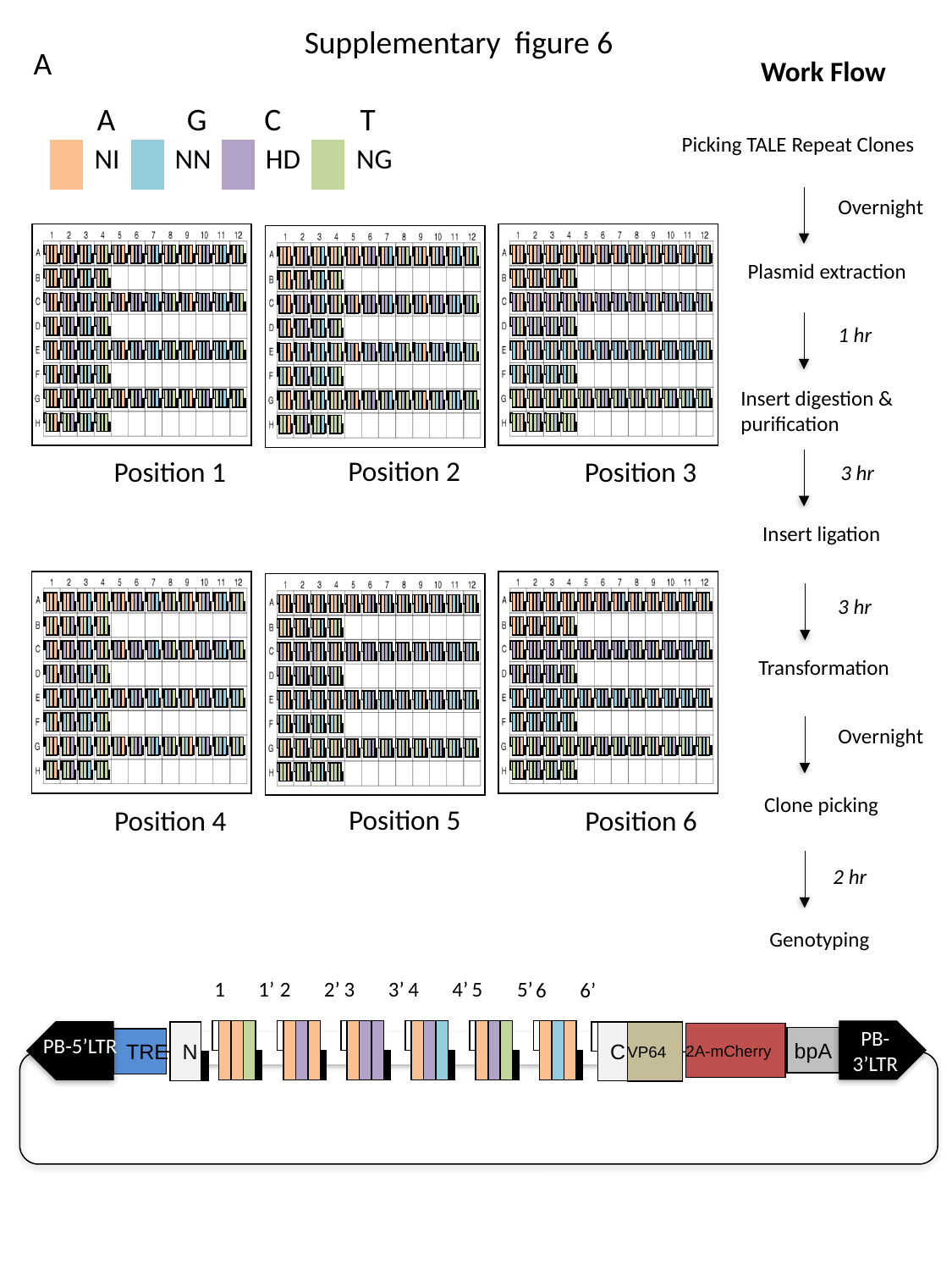

Supplementary figure 6
A
Work Flow
A G C T
Picking TALE Repeat Clones
| | NI | | NN | | HD | | NG |
| --- | --- | --- | --- | --- | --- | --- | --- |
Overnight
Plasmid extraction
1 hr
Insert digestion &
purification
Position 2
Position 1
Position 3
 3 hr
Insert ligation
Position 5
Position 4
Position 6
 3 hr
Transformation
Overnight
Clone picking
2 hr
Genotyping
1
1’
2
2’
3
3’
4
4’
5
5’
6
6’
PB-3’LTR
PB-5’LTR
N
C
VP64
2A-mCherry
bpA
TRE

## Slide 7
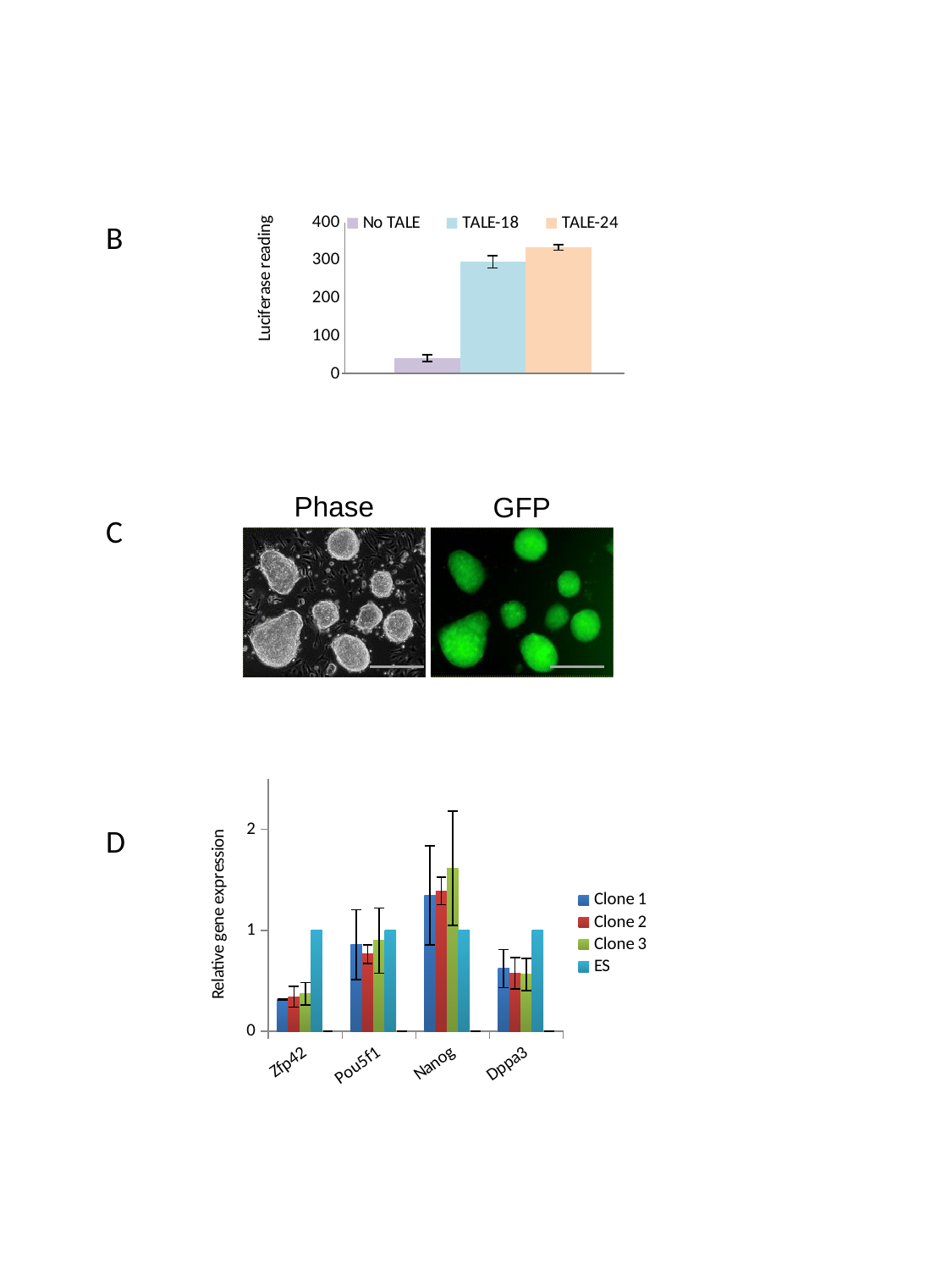

### Chart
| Category | | | |
|---|---|---|---|B
Phase
GFP
C
### Chart
| Category | Clone 1 | Clone 2 | Clone 3 | ES | MEFs |
|---|---|---|---|---|---|
| Zfp42 | 0.317102798057045 | 0.343058801387789 | 0.371284185441572 | 1.0 | 0.000184297187493676 |
| Pou5f1 | 0.859059910272389 | 0.764582070255825 | 0.898954218538405 | 1.0 | 0.00252957113785387 |
| Nanog | 1.347234686611995 | 1.390512189171515 | 1.615103401517305 | 1.0 | 0.002131566205991 |
| Dppa3 | 0.622545387910235 | 0.57619560646306 | 0.56371044861185 | 1.0 | 0.00011585809725495 |D
