# Supplementary material for: Comparison of TALE designer transcription factors and the CRISPR/dCas9 in regulation of gene expression by targeting enhancers
Source: Nucleic Acids Res. 2014 Sep 15;42(20):e155. doi: 10.1093/nar/gku836 (PMC4227760; doi:10.1093/nar/gku836)
Supplement: SUPPLEMENTARY DATA [file supp_gku836_nar-01182-met-h-2014-File005.docx]

**Table S1. Primers used for TALE repeat assembly, genomic DNA PCR and RT-PCR.**

| Primer | Primer sequence | Purpose |
| --- | --- | --- |
| TAL-F1 | ATATAGATGCCGTCCTAGCGcgtctcCTGACCCCAGAGCAGGTCGTGG | dTF assembling |
| TAL-R1 | TCTTATCGGTGCTTCGTTCTggtctcTGAGTCCGTGCGCTTGGCAC | dTF assembling |
| TAL-F2-2 | TGCTCTTTATTCGTTGCGTCggtctcGACTCACCCCAGAGCAGGTCGTG | dTF assembling |
| TAL-R2 | TCTTATCGGTGCTTCGTTCTggtctcTGAGGCCGTGCGCTTGGCAC | dTF assembling |
| TAL-F3-2 | TGCTCTTTATTCGTTGCGTCggtctcGCCTCACCCCAGAGCAGGTCGTG | dTF assembling |
| TAL-R4-3 | AAGTATCTTTCCTGTGCCCAcgtctcTTAAGCCGTGCGCTTGGCAC | dTF assembling |
| TAL-F5 | ATATAGATGCCGTCCTAGCGcgtctcGCTTAACCCCAGAGCAGGTCGTG | dTF assembling |
| TAL-F6-2 | TGCTCTTTATTCGTTGCGTCggtctcGACTCACCCCAGAGCAGGTCGTG | dTF assembling |
| TAL-R6 | TCTTATCGGTGCTTCGTTCTggtctcTGAGGCCGTGCGCTTGGCAC | dTF assembling |
| TAL-F7-2 | TGCTCTTTATTCGTTGCGTCggtctcGCCTCACCCCAGAGCAGGTCGTG | dTF assembling |
| TAL-R8-2 | AAGTATCTTTCCTGTGCCCAcgtctcTGAGCCCGTGCGCTTGGCAC | dTF assembling |
| TAL-F9-3 | ATATAGATGCCGTCCTAGCGcgtctcGGCTCACCCCAGAGCAGGTCGTG | dTF assembling |
| TAL-R9-3 | TCTTATCGGTGCTTCGTTCTggtctcTGAGTCCGTGCGCTTGGCAC | dTF assembling |
| TAL-F10-2 | TGCTCTTTATTCGTTGCGTCggtctcGACTCACCCCAGAGCAGGTCGTG | dTF assembling |
| TAL-R10 | TCTTATCGGTGCTTCGTTCTggtctcTGAGGCCGTGCGCTTGGCAC | dTF assembling |
| TAL-F11-2 | TGCTCTTTATTCGTTGCGTCggtctcGCCTCACCCCAGAGCAGGTCGTG | dTF assembling |
| TAL-R12-2 | AAGTATCTTTCCTGTGCCCAcgtctcTCAGCCCGTGCGCTTGGCAC | dTF assembling |
| TAL-F13 | ATATAGATGCCGTCCTAGCGcgtctcCGCTGACCCCAGAGCAGGTCGTG | dTF assembling |
| TAL-R13-2 | AAGTATCTTTCCTGTGCCCAcgtctcTCAGACCGTGCGCTTGGCAC | dTF assembling |
| TAL-F14 | ATATAGATGCCGTCCTAGCGcgtctcCTCTGACCCCAGAGCAGGTCGTG | dTF assembling |
| TAL-R14-2 | AAGTATCTTTCCTGTGCCCAcgtctcTGAGTCCGTGCGCTTGGCAC | dTF assembling |
| TAL-F15 | ATATAGATGCCGTCCTAGCGcgtctcCGTTGACCCCAGAGCAGGTCGTG | dTF assembling |
| TAL-R15 | AAGTATCTTTCCTGTGCCCAcgtctcTCAACCCGTGCGCTTGGCAC | dTF assembling |
| TAL-F-assem | ATATAGATGCCGTCCTAGCG | dTF assembling |
| TAL-R-assem | AAGTATCTTTCCTGTGCCCA | dTF assembling |
| NF-5.6 | TACTGCCTGGCTGTGTGTGGGTGC | Nanog ChIP-qPCR |
| NR-5.6 | AGCTCAGGCCCACAAAGCAGTTGGAGC | Nanog ChIP-qPCR |
| NF-5 | ACCTGTCCCTAGTCCCCGCTCCTTT | Nanog ChIP-qPCR |
| NR-5 | TGGCTGGTAGCCAAAAGGCAGGCT | Nanog ChIP-qPCR |
| NF-4.6 | TGACTCCGTGGACCCAGAGGCAAGT | Nanog ChIP-qPCR |
| NR-4.6 | AACCCTAGGTGTGTCCCAAGGGCGA | Nanog ChIP-qPCR |
| TyrF | GCTTCTTCATCCTGCTGGTC | Nanog ChIP-qPCR |
| TyrR | GGGAGCCATTCTCATTCAAA | Nanog ChIP-qPCR |
| GCKSF | ACAGCTTCGAAACTCTGGTG | GCKS qPCR |
| GCKSR | GAGAAGGACGGGAGCAGAG | GCKS qPCR |

BsmBI: cgtctcN, BsaI: ggtctcN.

**Table S2. Prime pairs used in monomer PCR for library assembling**

| Position | 1 | 2 | 3 | 4 | 5 | 6 | 7 | 8 | 9 |
| --- | --- | --- | --- | --- | --- | --- | --- | --- | --- |
| Primer pairs | F1  R1 | F2-2  R2 | F3-2  R4-3 | F5  R5 | F6  R6 | F7-2  R8-2 | F9-3  R9-3 | F10-2  R10 | F11-2  R12-2 |
| Position | 10 | 11 | 12 | 13 | 14 | 15 | 16 | 17 | 18 |
| Primer pairs | F13  R1 | F2-2  R2 | F3-2  R13-2 | F14  R5 | F6  R6 | F7  R15 | F15  R9-3 | F10-2  R10 | F11-2  R14-2 |

**Table S3. Applied Bioscience predesigned probes for real-time RT-PCR of mouse genes**

| Predesigned qPCR assays | Target | Applied Bioscience gene name |
| --- | --- | --- |
| Mm02384862_g1 | *Nanog* | Nanog homeobox |
| Mm03053975_g1 | *Zfp42* | Zinc finger protein 42 |
| Mm00836373_g1 | *Dppa3* | Developmental pluripotency associated 3 |
| Mm99999915_g1 | *Gapdh* | Mouse GADPH endogenous control |

**Table S4. DNA sequences bound by TALE and CRISPR.**

| Locus sites | CRISPRTarget sequence | TALE Target sequence |
| --- | --- | --- |
| Oct4, site 1 | CACAGCTCGGGACCAGGCTAGGG | TCGGGACCAGGCTAGGGCAC |
| Oct4, site 2 | TGAATACAGACAGGACTGCTGGG | TACAGACAGGACTGCTGGGC |
| Oct4, site 3-1 | GGGAGGAACTGGGTGTGGGGAGG | TAGCCCGACCCTGCCCCTCC |
| Oct4, site 3-2 | CCGACCCTGCCCCTCCCCCCAGG | TAGCCCGACCCTGCCCCTCC |
| Oct4, Site3-2 | CCCTGCCCCTCCCCCCAGGGAGG | TAGCCCGACCCTGCCCCTCC |
| Oct4, site 4 | CCTTCCTTAATCTGCTATTGAGG | TCCCTCTCGTCCTAGCCCTT |
| Nanog, Site1 | GACGGGTCTCCAGTAGTTCGAGG | TCAGAGGCAGACGGGTCTCC |
| Nanog, Site2 | ACAGGAATGGGGGTTGGGGAGGG | TCCCTCCCCAACCCCCATTC |
| Nanog, Site3 | TCAAACCGCTTCCCTGGATAAGG |  |
| Nanog, Site4 | TTAGTGTCTTTGAGAGTCTCTGG |  |
| Nanog, Site5 | TGGTACAGGGCGCACAGCTCCGG |  |
| Nanog, Site6 | GTCTGCTTAGGAGACGGCGATGG |  |
| gRNA control | TCAATTTAGTTACCTCACTATGG |  |
